# The role of G-protein-coupled membrane estrogen receptor in mouse Leydig cell function—in vivo and in vitro evaluation

**DOI:** 10.1007/s00441-018-2861-7

**Published:** 2018-06-06

**Authors:** M. Kotula-Balak, P. Pawlicki, A. Milon, W. Tworzydlo, M. Sekula, A. Pacwa, E. Gorowska-Wojtowicz, B. Bilinska, B. Pawlicka, J. Wiater, M. Zarzycka, J. Galas

**Affiliations:** 1 Department of Endocrinology, Institute of Zoology and Biomedical Research, Jagiellonian University in Kraków, Gronostajowa 9, 30-387 Krakow, Poland; 2 Department of Developmental Biology and Invertebrate Morphology, Institute of Zoology and Biomedical Research, Jagiellonian University in Kraków, Gronostajowa 9, 30-387 Krakow, Poland; 3 Department of Genetics and Evolutionism, Institute of Zoology and Biomedical Research, Jagiellonian University in Kraków, Gronostajowa 9, 30-387 Krakow, Poland; 4 Department of Cell Biology and Imaging, Institute of Zoology and Biomedical Research, Jagiellonian University in Kraków, Gronostajowa 9, 30-387 Krakow, Poland; 5 Medical Biochemistry, Jagiellonian University Medical College, Kopernika 7, 31-034 Krakow, Poland

**Keywords:** Leydig cell, G-coupled estrogen receptor, Estrogen receptors, estrogens, Ultrastructure

## Abstract

In this study, G-coupled estrogen receptor (GPER) was inactivated, by treatment with antagonist (G-15), in testes of C57BL/6 mice: immature (3 weeks old), mature (3 months old) and aged (1.5 years old) (50 μg/kg bw), as well as MA-10 mouse Leydig cells (10 nM/24 h) alone or in combination with 17β-estradiol or antiestrogen (ICI 182,780). In G-15-treated mice, overgrowth of interstitial tissue was found in both mature and aged testes. Depending on age, differences in structure and distribution of various Leydig cell organelles were observed. Concomitantly, modulation of activity of the mitochondria and tubulin microfibers was revealed. Diverse and complex GPER regulation at the mRNA level and protein of estrogen signaling molecules (estrogen receptor α and β; ERα, ERβ and cytochrome P450 aromatase; P450arom) in G-15 Leydig cells was found in relation to age and the experimental system utilized (in vivo and in vitro). Changes in expression patterns of ERs and P450arom, as well as steroid secretion, reflected Leydig cell heterogeneity to estrogen regulation throughout male life including cell physiological status.We show, for the first time, GPER with ERs and P450arom work in tandem to maintain Leydig cell architecture and supervise its steroidogenic function by estrogen during male life. Full set of estrogen signaling molecules, with involvement of GPER, is crucial for proper Leydig cell function where each molecule acts in a specific and/or complementary manner. Further understanding of the mechanisms by which GPER controls Leydig cells with special regard to male age, cell of origin and experimental system used is critical for predicting and preventing testis steroidogenic disorders based on perturbations in estrogen signaling.

## Introduction

Estrogen is a lipophilic hormone that easily dissolves in lipids, allowing it to diffuse into the plasma membrane, resulting in interactions with hydrophobic surfaces of proteins and other macromolecules. This allows estrogen to associate with receptors that may reside at the cell membrane or in the cytoplasm, thereby promoting a diverse array of biochemical actions with different kinetics, including acute (non-genomic) or long-lasting (genomic) actions. To date, estrogen receptors belonging to the two distinct receptor families have been described: estrogen receptors ERα and ERβ that are categorized as steroid hormone receptors and G-protein-coupled membrane estrogen receptor (GPER), a member of the G-protein receptor superfamily (Sharma and Prossnitz 2011; Sandner et al. 2014; Acconcia et al. 2016). ERs and GPER each participate in estrogen signaling and likely act in a coordinate manner. Each estrogen receptor type is considered unique and independent based on genetic, pharmacological, biological and biochemical evidence (that shows distinct physical and functional properties), as well as displays individual phenotypes in knockout mice (Couse and Korach 1999; Carreau et al. 2003; Gaudet et al. 2015). Moreover, these receptors localize to diverse subcellular environments, possess a unique binding characteristic and interact distinctly with selective ligands promoting specific responses.

In males, estrogen is produced by spermatogenic cells (of all stages) and somatic cells of the testis. Leydig cells are the major source of estrogen in the adult testis, while Sertoli cells synthesize the majority of estrogen in immature testis (Schulster et al. 2016). The production of estrogens from androgens is governed by cytochrome P450 aromatase within the endoplasmic reticulum of cells, which is expressed also under spatio-temporal control. P450 aromatase is responsible for catalyzing the series of reactions that lead to the irreversible conversion of testosterone and androstenedione into estradiol and estrone, respectively (Carreau et al. 2003). A proper balance between androgen and estrogen is fundamental for normal male reproductive development and function in both animals and humans. In various species including humans, a more significant estrogen concentration exists in the male reproductive tract and semen than in the serum (Hess 2003). Interestingly, in boars, the intratesticular estradiol level is higher than that in dams in estrus (Hoffmann et al. 2010). Regulation of testicular cells by estrogen shows both an inhibitory and a stimulatory influence, indicating an intricate symphony of dose-dependent and temporally sensitive modulation. In various tissues, estrogen controls growth, differentiation and proliferation/apoptosis migration of both normal and malignant cells (Barakat et al. 2016; Acconcia et al. 2016). Estrogen is also a metabolic hormone and it supervises the integrated physiology of tissues regulating lipid homeostasis (Shen and Shi 2015).

In the male gonad, inhibition of steroidogenic Leydig cell regeneration in ethane dimethylsulfonate-treated rats, after subsequent estradiol exposure, indicates Leydig cells self-regulate their number via estrogen modulation in a paracrine fashion of fetal Leydig cell quantity (Abney and Myers 1991). Furthermore, there is evidence suggesting that estradiol inhibits effect of lutropin (LH) on Leydig cells and excess estrogen reduces serum testosterone levels (Hess 2003). The subsequent decrease of testosterone in turn inhibits spermatogenic function (Atanassova et al. 1999). Studies have confirmed that, in the above processes, estrogen signaling via ERs localized in testicular cells in a species-, age- and physiological status-specific way takes place (for review see Hess 2000; Carreau et al. 2012). Of note, the unique presence of ERβ in the mitochondria reported since now in cells of human uterus, ovary, cardiomyocytes, rat primary neurons, human bone narrow neuroblast (SH-SY5Y) cells, lens epithelial cells, sperm, human breast cancer (MCF-7) cells, human non-small cell lung cancer (NSCLC) cells, human adenocarcinomic alveolar basal epithelial (A549) cells, human hepatoma (HepG2) cells, human osteosarcoma (SaOS-2 and 143B) cells and human retinal ganglion cells (for review see Liao et al. 2015), while ERs splice variants present on the cell membrane (Irsik et al. 2013) have undiscovered a contribution to this signaling.

Expression and function of GPER occur independently of the two nuclear ERs. GPER has a high affinity for estrogens but only a limited binding capacity with single estrogen-binding sites (Lazari et al. 2009; Carreau and Hess 2010; Li et al. 2015). Compared to ERs, its binding affinity for 17β-estradiol is considerably lower and the association and dissociation rates are very rapid.

Using a *Gper*-lacZ reporter mouse, extensive expression of testicular GPER was demonstrated (Isensee et al. 2009). Until recently, function of GPER in testicular cells was only partially known. GPER expression was found in a mouse spermatogonia cell line GC-1 (Sirianni et al. 2008), in adult rat pachytene spermatocytes and round spermatids (Chimento et al. 2010, 2011) highlighting a role for this receptor in spermatogenesis. Moreover, GPER expression has been currently demonstrated in Sertoli cells, highlighting their multiple function in seminiferous tubule physiology (Lucas et al. 2010, 2011). Sandner et al. (2014) observed an inverse relationship of GPER expression and fertility in peritubular cells of monkey and human. Using qPCR, Fietz et al. (2014, 2016) showed GPER expression in Leydig cells and Sertoli cells of human testis. Expression level in Leydig cells was the highest when compared to Sertoli cells and human breast cancer (MCF-7) cells. Unfortunately, GPER protein was not analyzed (due to problem with antibody). Thus, in primate peritubular cells, GPER mediates estrogen action in both, testis in health and disease. Our previous studies demonstrated the presence of GPER in Leydig cells of bank voles with various sex hormones levels; however, receptor expression was not altered in regard to normal or physiologically decreased intratesticular estrogen concentration (Zarzycka et al. 2016). The involvement in GPER action second messengers such as protein kinase A and extracellular signals-regulated kinase, as well as Ca2+, cAMP, cGMP and metalloproteinase 9, was reported too. Nonetheless, the role of GPER for Leydig cell morpho-functional status needs further detailed attention.

It has been shown that GPER regulates the proliferative and apoptotic pathways involved in spermatogenesis (Prossnitz and Barton 2011) but there are no data on steroidogenic testis function. No clear developmental or functional defects in the reproductive organs of GPER knockout male mice were reported (Mårtensson et al. 2009; Otto et al. 2009). On the other hand, GPER knockouts were moderately obese with larger adipocyte size indicating lipid homeostasis disturbances. It should be mentioned here that although there are still controversies on ERβ knockout mouse fertility, ERα knockouts exhibit various reproductive organ defects (Krege et al. 1998; Antal et al. 2008; Lee et al. 2009). In efferent ducts, fluid absorption is altered due to aquaporin 1 and carbonic anhydrase dysfunction (Hess 2000). The role of GPER in the male reproductive system is complex and regulated at multiple levels, thus requiring further in-depth investigation. This study aims to better understand the involvement of GPER in Leydig cell function via examination of the morpho-functional and secretion status of these cells. Moreover, the interaction between GPER and ERs together with P450 aromatase is a crucial goal of the present study. To our knowledge, this is the first in vitro and in vivo study on the importance of GPER in testicular Leydig cell biology.

## Materials and methods

### Animals and treatments

Male mice (C57BL/6) 3 weeks old (*n* = 10), 3 months old (*n* = 10) and 1.5 years old (*n* = 10) were obtained from the Department of Genetics and Evolution, Institute of Zoology and Biomedical Research, Jagiellonian University, Kraków. Animals were maintained on 12 h dark-light (250 lx at cages level) cycle with stable temperature condition (22 °C), relative humidity of 55 ± 5% and free access to water and standard pelleted diet (LSM diet, Agropol, Motycz, Poland). Animals were killed by cervical dislocation. The use of the animals was approved by the National Commission of Bioethics at the Jagiellonian University in Krakow, Poland (No. 151/2015).

Mice from various age groups were allotted into experimental groups (each group including 5 animals); and control (Cont.) and treated receiving selective GPER receptor antagonist [(3aS*,4R*,9bR*)-4-(6-bromo-1,3-benzodioxol-5-yl)-3a,4,5,9b-3*H*-cyclopenta[*c*]quinolone; G-15] (Tocris Bioscience, Bristol, UK). G-15 was dissolved in DMSO and the stock solutions were kept at − 20 °C. Animals from the experimental groups were injected subcutaneously with freshly prepared solutions of G-15 (50 μg/kg bw) in phosphate buffered saline (six doses each dose injected every other day). Mice from control groups received vehicle only. Dose, frequency and time of G-15 administration were based on literature data (Dennis et al. 2009; An et al. 2014; Kang et al. 2015) and it was finally selected upon our preliminary study in mice in vivo (doses range between 5, 50, 100, 150, 200 μg/kg bw). Both testes of each individual of control and G-15-treated mice were surgically removed and were cut into small fragments. For histology and immunohistochemistry, tissue samples were fixed in 10% formalin and embedded in paraplast. Small pieces of the testicular tissue were immediately fixed in glutaraldehyde for transmission microscopy analysis or frozen in a liquid nitrogen and stored at − 80 °C for RNA isolation and determination of steroid hormones.

### Histology

For routine histology, hematoxylin-eosin staining was performed. The sections were examined under a Nikon Eclipse Ni-U microscope (Nikon, Tokyo, Japan). A tubus setting of 1.25, a × 10 ocular and a × 10 objective was used for the measurements. Detailed morphologic analysis was performed with the use of NIS-Elements software (Nikon, Tokyo, Japan), as previously described (Kotula-Balak et al. 2012). The area of the interstitium occupied by Leydig cells was determined in 40 random fields of vision (which corresponds to 17.7 mm^2^) for each animal from the control and treated groups. A mean was determined for control animals and those treated with G-15.

### Cell culture and treatments

The mouse Leydig cell line MA-10 was a generous gift from Dr. Mario Ascoli (University of Iowa, Iowa City, USA) and was maintained under standard technique (Ascoli 1981). Middle passages (p25-p28) of MA-10 cells were used for the study. The cells were grown in Waymouth’s media (Gibco, Grand Island, NY) supplemented with 12% horse serum and 50 mg/l of gentamicin at 37 °C in 5% CO_2_. Cells were plated overnight at a density of 1 × 10^5^ cells/ml per well. Morphological and biochemical properties of MA-10 cells were regularly checked by microscopic observation, analysis of proliferation (TC20 Bio-Rad automated cell counter), mycoplasma detection (MycoFluor™ Mycoplasma Detection Kit; ThermoFisher Scientific), qRT-PCR analysis of characteristic genes and ELISA measurements of secretion products according to cell line authentication recommendations of the Global Bioresource Center (ATCC).

Twenty-four hours before the experiments, the medium was removed and replaced with a medium without phenol red supplemented with 5% dextran-coated, charcoal-treated FBS (5% DC-FBS) to exclude estrogenic effects caused by the medium. Next, cells were treated with selective GPER receptor antagonist [(3aS*,4R*,9bR*)-4-(6-bromo-1,3-benzodioxol-5-yl)-3a,4,5,9b-3*H*-cyclopenta[*c*]quinolone; G-15] (Tocris Bioscience, Bristol, UK) freshly prepared 100 μM stock solution in dimethyl sulfoxide (DMSO) (Sigma-Aldrich) stored at − 20 °C. A stock concentration was subsequently dissolved in Waymouth’s media to final concentration of 10 nM. Cells were treated with G-15 alone or together with 17β-estradiol (Sigma-Aldrich; 10 mM) and ER antagonist ICI 182,780 (ICI; Faslodex, Sigma–Aldrich, St. Louis, MO, USA; 10 μM) freshly prepared in ethanol, for 24 h. Dose of G-15 was based on literature data (Dennis et al. 2011; Bertrand et al. 2015; Carnesecchi et al. 2015; Treen et al. 2016) and it was finally selected upon our preliminary study (dose range 1, 10, 100 nM). Doses of E2 and ICI were based on our previous studies (Kotula-Balak et al. 2013; Pardyak et al. 2016; Milon et al. 2017). Control cells were treated with DMSO or ethanol or both together (final conc. 0.1%). We performed microscopic analysis of ultrastructure, as well as mitochondria activity and cytoskeleton structure of Leydig cells. Culture media were frozen in − 20 °C for steroid hormone level determination.

### Ultrastructure

The fixation procedure described below was based on the protocols proposed by Russell and Burguet (1977). The modification developed in our labs had important advantages: it improved the quality of fixation and enhanced the contrast of plasma membrane and the organelles. Briefly, Leydig cells in vitro and dissected testes (control and G-15-treated) were immersed in ice-cold pre-fixative containing 2% formaldehyde and 2.5% glutaraldehyde in 0.1 M phosphate buffer, pH 7.3. The tissues were then rinsed and post-fixed in a mixture of 2% osmium tetroxide and 0.8% potassium ferrocyanide in the same buffer for 30 min at 4 °C. The material was embedded in Glycid Ether 100 resin (Serva, Heidelberg, Germany). Semi-thin sections (0.7 μm thick) were stained with 1% methylene blue and examined under a Leica DMR (Wetzlar, Germany) microscope. Prior to embedding, small (3–5 mm) pieces of testicular tissue were carefully oriented in the mold to obtain accurate cross-sections of the tubules. Ultrathin sections (80 nm thick) were contrasted with uranyl acetate and lead citrate and analyzed with a JEOL 2100 HT (Japan) TEM.

### Mitochondrial activity and cytoskeleton structure

Leydig cells (control, G-15, and estradiol-treated) were grown on coverslips (Ø12 mm; Menzel Gläser, Germany) and used as live. For mitochondrial activity analysis, MitoTracker™ Orange CMTMRos (Thermo Fisher Scientific) was applied. Preparation of dye stock solution (1 mM in DMSO) and preformation of staining was prepared based on manufacturer’s protocol. For tubulin filaments labeling Tubulin Tracker™ Oregon Green ® (Thermo Fisher Scientific), 500 μM in DMSO according to manufacturer’s protocol was used.

Stained cells were analyzed in a LSM 510 META confocal system with a Zeiss Axiovert 200M inverted microscope (Carl Zeiss GmbH, Jena, Germany). To evaluate the intensity of fluorescence quantitatively, digital images were obtained and analyzed using public domain ImageJ software (National Institutes of Health, Bethesda, Maryland, USA). The intensity of fluorescence was calculated using the formula described by Smolen (1990) and expressed as relative fluorescence in arbitrary units. Results of 20–30 separate measurements were expressed as mean ± SD.

### RNA isolation, reverse transcription

Total RNA was extracted from control and G-15-treated mouse testes and Leydig cells using TRIzol® reagent (Life Technologies, Gaithersburg, MD, USA) according to the manufacturer’s instructions. To remove contaminating DNA and DNase from RNA preparations, the RNA samples were incubated with reagents from the TURBO DNA-free™ Kit (Ambion, Austin, TX). The yield and quality of the RNA were assessed using a NanoDrop ND2000 spectrophotometer (Thermo Scientific, Wilmington, DE, USA). Samples with a 260/280 ratio of 1.95 or greater and a 260/230 ratio of 2.0 or greater were used for analysis. Total cDNA was prepared using High-Capacity cDNA Reverse Transcription Kit (Applied Biosystems, Carlsbad, CA, USA) according to the manufacturer’s instructions.

The purified total RNA was used to generate total cDNA. A volume equivalent to 1 μg of total RNA was reverse transcribed using the High-Capacity cDNA Reverse Transcription Kit (Applied Biosystems, Carlsbad, CA, USA) according to the manufacturer’s instructions. Total cDNA was prepared in a 20-μL volume using a random primer, dNTP mix, RNase inhibitor and reverse transcriptase (RT). Parallel reactions for each RNA sample were run in the absence of RT to assess genomic DNA contamination. RNase-free water was added in place of the RT product.

### Real-time quantitative RT-PCR

Real-time RT-PCR was performed using the StepOne Real-Time PCR system (Applied Biosystems) and optimized standard conditions as described previously by Kotula-Balak et al. (2012, 2013). Based on the gene sequences in the Ensembl database, primer sets were designed using Primer3 software (Table 1). Selected primers were synthesized by the Institute of Biochemistry and Biophysics, Polish Academy of Science (Warsaw, Poland).

**Table 1:** Sequences of forward and reverse primers

Genes	Primers (5′–3′) GCCTCT	Product size (bp)	Annealing temperature (°C)	Cycles	References
*GPER*	5′- CTGGACGAGCAGTATTACGATATC - 3′ 5′- TGCTGTACATGTTGATCTG - 3′	295	62	35	http://www.ensembl.org (ENSMUSG00000053647)
*P450arom*	5′- CCCCTGGACGAAAGTTCTATTG - 3′ 5′- CAGCGAAAATCAAATCAGTTGC - 3′	238	62	35	http://www.ensembl.org (ENSMUSG00000032274)
*ERα*	5′- GCGCAAGTGTTACGAAGTGG - 3′ 5′- AAGCCTGGCACTCTCTTTGC - 3′	375	60	40	http://www.ensembl.org (ENSMUSG00000019768)
*ERβ*	5′- TCTGTGTGAAGGCCATGATC - 3′ 5′- GCAGATGTTCCATGCCCTTG - 3′	237	60	40	http://www.ensembl.org (ENSMUSG00000021055)
*TUBa1α*	5′- CGGAACCAGCTTGGACTTCTTTCCG - 3′ 5′- GGAACTGGCTCTGGCTTCACC - 3′	321	60	40	http://www.ensembl.org (ENSMUST00000134214)

*GPER* G-coupled membrane estrogen receptor, *P450arom* cytochrome P450 aromatase, *ERα* estrogen receptor alpha, *ERβ* estrogen receptor beta, *TUBa1α* tubulin a1α

To calculate the amplification efficiency, serial cDNA dilution curves were produced for all genes (Pfaffl 2001). A graph of threshold cycle (Ct) versus log10 relative copy number of the sample from a dilution series was produced. The slope of the curve was used to determine the amplification efficiency: %E = (10^–1/slope−1^) × 100. All PCR assays displayed efficiency between 94 and 104%.

Detection of amplification products for *GPER*, *ERα*, *ERβ* and *P450arom* and for the reference gene Tubulin a1α (*Tuba1α*), was performed with 10 ng cDNA, 0.5 μM primers and SYBR Green master mix (Applied Biosystems) in a final volume of 20 μL. Amplifications were performed as follows: 55 °C for 2 min, 94 °C for 10 min, followed by annealing temperature for 30 s (Table 1) and 45 s 72 °C to determine the cycle threshold (Ct) for quantitative measurement as described previously (Kotula-Balak et al. 2013). To confirm amplification specificity, the PCR products from each primer pair were subjected to melting curve analysis and subsequent agarose gel electrophoresis. In all real-time RT-PCR reactions, a negative control corresponding to RT reaction without the reverse transcriptase enzyme and a blank sample were carried out (not shown in all figures). All PCR products stained with Midori Green Stain (Nippon Genetics Europe GmbH, Düren, Germany) were run on agarose gels. Images were captured using a Bio-Rad Gel Doc XR System (Bio-Rad Laboratories, Hercules, CA, USA). *GPER*, *ERα*, *ERβ* and *P450arom* mRNA expressions were normalized to the *Tuba1α* mRNA (tested with other references genes: GAPDH and β-actin in a pilot study) (relative quantification, RQ = 1) with the use of the 2^−ΔΔCt^ method, as previously described by Livak and Schmittgen (2001).

Three independent experiments were performed, each in triplicate with tissues prepared from different animals.

### Immunohistochemistry, immunocytochemistry and immunofluorescence

To optimize immunohistochemical staining, testicular sections both control and G-15-treated were immersed in 10 mM citrate buffer (pH 6.0) and heated in a microwave oven (2 × 5 min, 700 W). Thereafter, sections were immersed sequentially in H_2_O_2_ (3%; *v*/*v*) for 10 min and normal goat serum (5%; *v*/*v*) for 30 min that were used as blocking solutions. Afterwards, sections were incubated overnight at 4 °C with primary antibodies listed in Table 2. Next, respective biotinylated antibodies (anti-rabbit, anti-goat, and anti-mouse IgGs; 1: 400; Vector, Burlingame CA, USA) and avidin-biotinylated horseradish peroxidase complex (ABC/HRP; 1:100; Dako, Glostrup, Denmark) were applied in succession. Bound antibody was visualized with 3,3′-diaminobenzidine (DAB) (0.05%; *v*/*v*; Sigma-Aldrich) as a chromogenic substrate. Control sections included omission of primary antibody and substitution by irrelevant IgG. Thereafter, sections were washed and were slightly counterstained with Mayer’s hematoxylin and mounted using DPX mounting media (Sigma–Aldrich).

**Table 2:** Primary antibodies used for immunocyto-, immunohisto- and immunofluorescence

Antigen	Host species of primary antibodies	Vendor	Dilution	Host species of secondary antibodies	Vendor
GPER	Rabbit	Abcam cat.no. ab39742	1:50 (ICC) 1:250 (IHC)	Biotinylated goat anti-rabbit (ICC, IHC)	Vector Laboratories BA-1000
			1:100 (IF)	Alexa Fluor 488 goat anti-rabbit (IF)	Thermo Fisher Scientific A-11008
P450arom	Rabbit	Santa Cruz Biotechnology cat.no. sc-30086	1:100 (ICC) 1:500 (IHC)	Goat anti-rabbit	Vector Laboratories BA-1000
ERα	Rabbit	Abcam cat.no. ab75635	1:20 (ICC) 1:100 (IHC)	Goat anti-rabbit	Vector Laboratories BA-1000
ERβ	Rabbit	Abcam cat.no. ab3576	1:20 (ICC) 1:50 (IHC)	Goat anti-rabbit	Vector Laboratories BA-1000

*GPER* G-coupled membrane estrogen receptor, *P450arom* cytochrome P450 aromatase, *ERα* estrogen receptor alpha, *ERβ* estrogen receptor beta

Immunocytochemistry or immunofluorescence labeling was performed on Leydig cells (prepared as previously mentioned). Cells were fixed using 4% paraformaldehyde for 5 min or absolute methanol for 7 min followed by acetone for 4 min both at − 20 °C respectively. Next, only cells for immunocytochemistry were rinsed in TBS containing 0.1% Triton X-100. Nonspecific binding sites were blocked with 5% normal goat serum for 30 min. Thereafter, cells were incubated overnight at 4°C in a humidified chamber in the presence of primary antibodies listed in Table 2. On the next day, biotinylated antibody goat anti-rabbit (1:400; Vector Laboratories) or Alexa Fluor 488 goat anti-rabbit antibody (1:100; Invitrogen, Co., Carlsbad, CA, USA) was applied for 45 and 60 min, respectively. After each step in these procedures, cells were carefully rinsed with TBS; the antibodies were also diluted in TBS buffer. The staining for the light microscopy was developed using ABC/HRP complex for 30 min followed by DAB. Thereafter, cells were washed and were slightly counterstained with Mayer’s hematoxylin and mounted using DPX mounting media (Sigma–Aldrich). Cells were examined with a Leica DMR microscope (Leica Microsystems, Wetzlar, Germany). Fluorescent staining was protected from light and cells were mounted with Vectashield mounting medium (Vector Labs) with 40,6-diamidino-2-phenylindole (DAPI) or without DAPI and next examined with an epifluorescence microscope Leica DMR (Leica Microsystems) equipped with appropriate filters.

The whole procedure was described in detail elsewhere (Kotula-Balak et al. 2013; Zarzycka et al. 2016; Pawlicki et al. 2017). Experiments were repeated three times.

### Radioimmunoassay

Culture media (100 μl) of control and G-15, E2, ICI-treated Leydig cells were analyzed for progesterone content using the radioimmunological technique described elsewhere (Abraham et al. 1971). Progesterone level was determined using [1,2,6,7-^3^H]-progesterone (Amersham International plc), specific activity 96 Ci/mmol, as a tracer and an antibody raised in a sheep against 11β-hydroxyprogesterone succinyl-bovine serum albumin (BSA), (a generous gift from Prof. Brian Cook, University Glasgow, Scotland, UK). Progesterone assay was validated by demonstrating parallelism between serial dilutions of culture media and standard curve. It cross-reacted with pregnenolone (1.8%), corticosterone (1.5%), 17α-hydroxyprogesterone (only 0.8%) and testosterone (only 0.12%). Binding of four related steroids such as 20α-dihydroprogesterone, 20β-dihydroprogesterone, 17α-hydroxy-20 β-dihydroprogesterone, 17α and 20α-hydroxyprogesterone and other steroids was below 0.01%. Coefficients of variation within and between assays were 5.0 and 9.8%, respectively.

To determine and testosterone and estradiol level in testicular homogenates of control and G-15-treated mouse testes, the radioimmunological technique described elsewhere (Hotchkiss et al. 1971; Dufau et al. 1972; Pawlicki et al. 2017) was used. Testosterone levels were assessed using [1,2,6,7-^3^H]-testosterone (specific activity 110 Ci/mmol; American Radiolabeled Chemicals, Inc.) as a tracer and rabbit antibody against testosterone-3-0-CMO:BSA (a gift from Dr. B. Ričařova, Institute of Radiology, Czech Academy of Sciences, Prague, Czech Republic). The lower limit of sensitivity was 5 pg. Cross-reaction of this antibody was 18.3% with dihydrotestosterone, 0.1% with androstenedione and less than 0.1% with other major testis steroids. Coefficients of variation within and between assays were below 5.0 and 9.7%, respectively.

Estradiol concentrations were measured using [2,4,6,7-^3^H]-estradiol (specific activity 81 Ci/mmol: American Radiolabeled Chemicals, Inc.) as a tracer and rabbit antibody against estradiol-17-O-carboxymethyloxime: BSA (a gift from Institute of Pharmacology, Polish Academy of Sciences, Krakow, Poland). The lower limit of sensitivity of the assays was 5 pg. Cross-reaction was 1% with keto-oestradiol-17b, 0.8% with estrone, 0.8% with estriol, 0.01% with testosterone and less than 0.1% with major ovarian steroids. Coefficients of variation within and between assays were below 4 and 7.5%, respectively. Assays were validated by demonstrating parallelism between serial dilutions of culture media and standard curve. Coefficients of variation within and between each assay were 7.6 and 9.8%, respectively. The recovery of unlabeled steroids was also assessed (never less than 90%). In addition to monitoring intra-assays and inter-assays, assay quality control was assessed by control samples representing low, medium and high concentrations of measured hormones. Samples (each in triplicate with tissues prepared from different animals) were counted in a scintillation counter (LKB 1209 RACKBETA LKB; Turku, Finland). The concentrations of sex steroids were calculated as pg/10^5^ cells.

### Statistical analysis

Each variable was tested by using the Shapiro-Wilk *W* test for normality. Homogeneity of variance was assessed with Levene’s test. Since the distribution of the variables was normal and the values were homogeneous in variance, all statistical analyses were performed using one-way analysis of variance (ANOVA) followed by Tukey’s *post hoc* comparison test to determine which values differed significantly from controls. The analysis was made using Statistica software (StatSoft, Tulsa, OK, USA). Data were presented as mean ± SD. Data were considered statistically significant at *p* < 0.05.

## Results

### GPER mRNA level and protein localization in mouse testes and Leydig cells

**Fig. 1:**
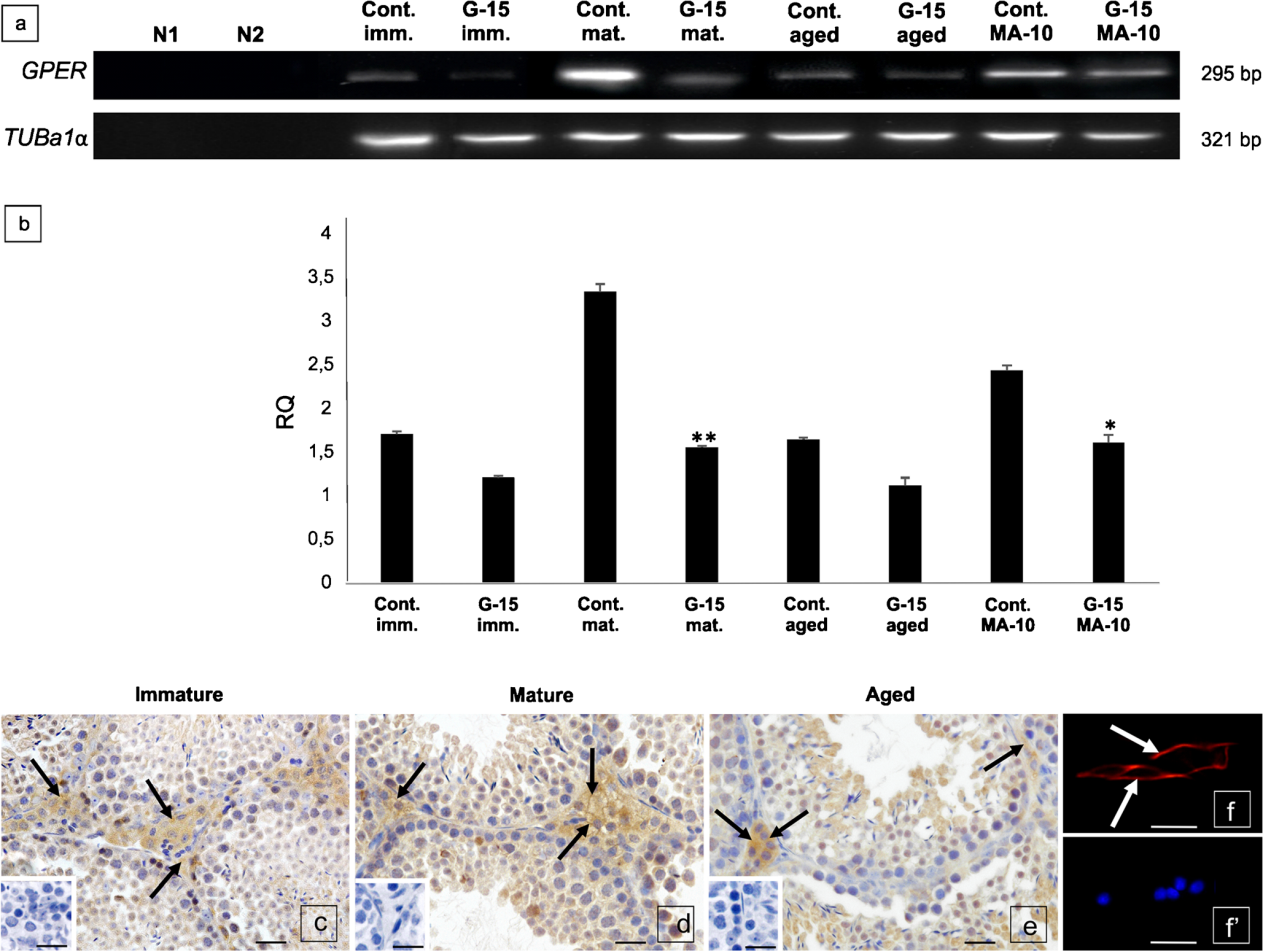
GPER mRNA level and protein localization in mouse testes and Leydig cells. (**a**–**f**’). **a** Representative gel electrophoresis of qualitative expression, (line N1-negative control without complementary DNA template, line N2-negative control without nonreverse transcribed RNA), **b** and relative quantification (RQ) of mRNA for GPER in mouse testes; immature, mature, aged [control and G-15 (50 μg/kg bw)-treated] and mouse MA-10 Leydig cells [control and G-15 (10 nM)-treated]. RQ is expressed as means ± SD. Asterisks show significant differences between control and G-15-treated testes/cells. Values are denoted as *p < 0.05 **p < 0.01. From each animal, three mRNA samples were analyzed. (**c**–**f**’) Representative microphotographs of cellular localization of GPER in membrane and cytoplasm of Leydig cells of immature (**c**), mature (**d**) and aged (**e**) mouse testes and in membrane of mouse MA-10 Leydig cells (**f**) (arrows). Immunostaining with DAB and counterstaining with hematoxylin (**c**–**e**). Scale bars represent 15 μm. Staining was performed on testicular serial sections from at least three animals from each group. Immunofluorescence with DAPI (**f**, **f**’). Scale bars represent 20 μm. Immunoreaction was performed on Leydig cell cultures in triplicate. Inserts in (**c**–**e**) and (**f**’)—negative controls—no immunostaining is visible when the primary antibody is omitted

Depending on animal age, as well as Leydig cell of origin (mouse testis or tumor mouse Leydig cell line), differences in mRNA level were found (Fig. 1a, b). G-15 treatment decreased expression of GPER in all treated age groups, with a significant decrease in mature animals (*p* < 0.01). In MA-10 Leydig cells treated with G-15, a significant decrease (*p* < 0.05) was observed as well.

In immature, mature and aged testis sections, membrane-cytoplasmic localization of GPER was found (Fig. 1c–e). In MA-10 Leydig cells, GPER was exclusively localized to the membrane (Fig. 1f).

### Effect of GPER blockage on mouse testis histology and Leydig cells morphology

**Fig. 2:**
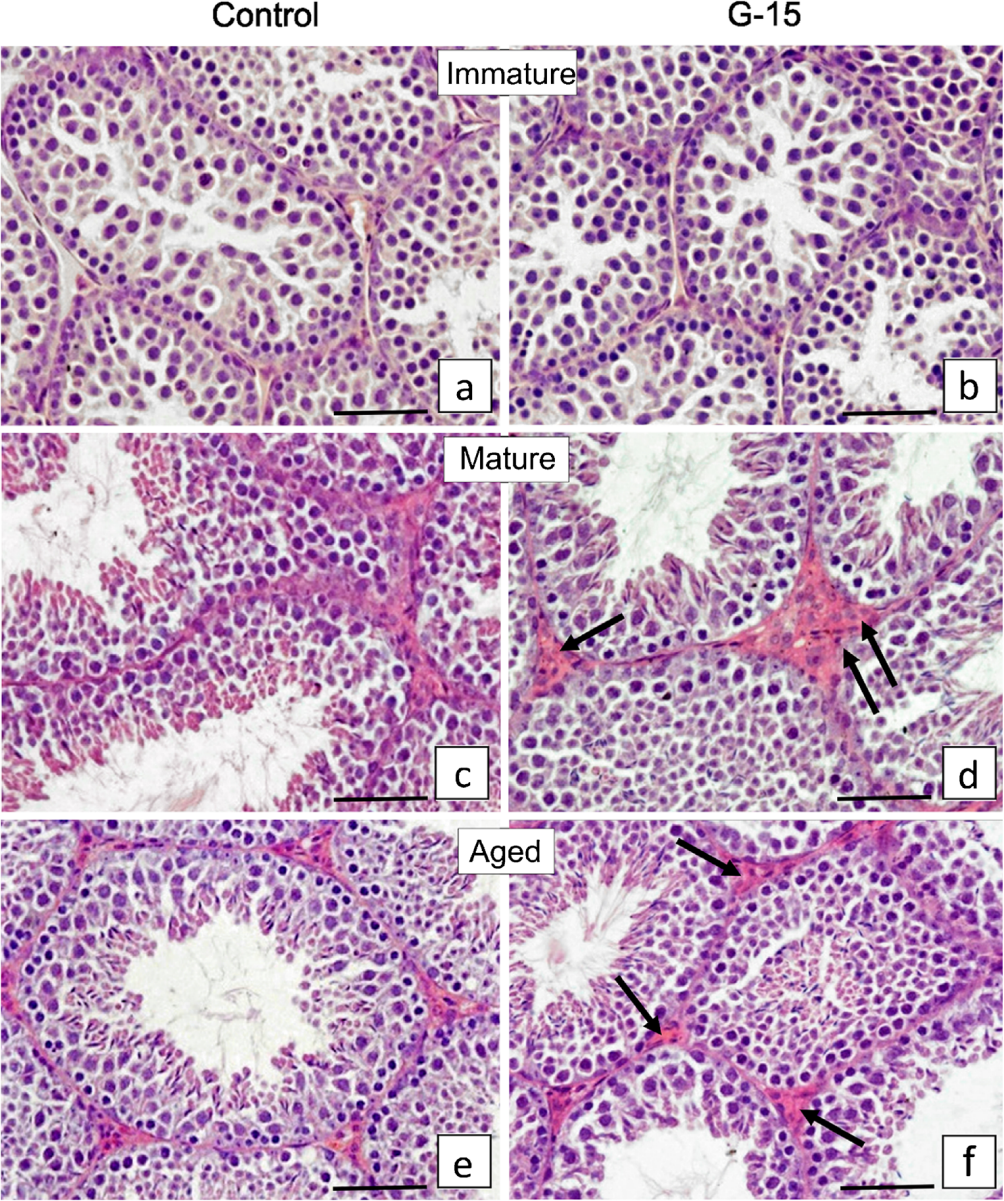
Effect of GPER blockage on mouse testis histology and Leydig cells morphology (**a–f**). Representative microphotographs of (**a**, **c**, **e**) control and (**b**, **d**, **f**) G-15 (50 μg/kg bw)-treated mouse testes. (**a**, **b**) Immature (**c**, **d**) mature and (**e**, **f**) aged mouse testicular sections. Hematoxylin-eosin staining. Scale bars represent 15 μm. Staining was performed on testicular serial sections from at least three animals of each experimental group. Small clusters of Leydig cells and not active seminiferous tubules but with open lumens in a majority of tubules, in both control and G-15-treated immature males are observed (**a**, **b**). Full spermatogenesis in seminiferous tubules of both control and G-15 mature males (**c**, **d**). Leydig cells in small groups in control (**c**) but enlarged interstitial tissue with Leydig cells after exposure to G-15 (**d**) (arrows) is visible. Full spermatogenesis but not as active as in mature mice is observed in aged control and G-15 males (compare number of elongated spermatids in lumens of tubules **c**, **d** and **e**, **f**). Leydig cells surrounding seminiferous tubules and located in groups. Subtle differences in abundancy of interstitial tissue are seen between control and G-15 aged males (arrows) (**e**, **f**)

G-15 treatment exerted no effect on immature testis while a slight effect was observed in mature and aged mouse testis histology (Fig. 2a–f). In control and treated immature males, the seminiferous tubules filled up with spermatogenic cells and small clusters of Leydig cells were observed (Fig. 2a, b). In control and treated mature males, normal seminiferous tubules with full spermatogenesis were seen (Fig. 2c, d). In addition, overgrowth of interstitial tissue was visible when compared to control (2.21 ± 0.20 vs 1.97 ± 0.14 mm^2^). In both control and treated aged animals, a small number of elongated spermatids in lumens of tubules when compared to mature animals was observed (Fig. 2e, f and c, d). In treated males, when compared to controls, a slight increase in the interstitial tissue volume was revealed (2.69 ± 0.05* vs 2.06 ± 0.09 mm^2^).

### Effect of GPER blockage on Leydig cell ultrastructure

**Fig. 3:**
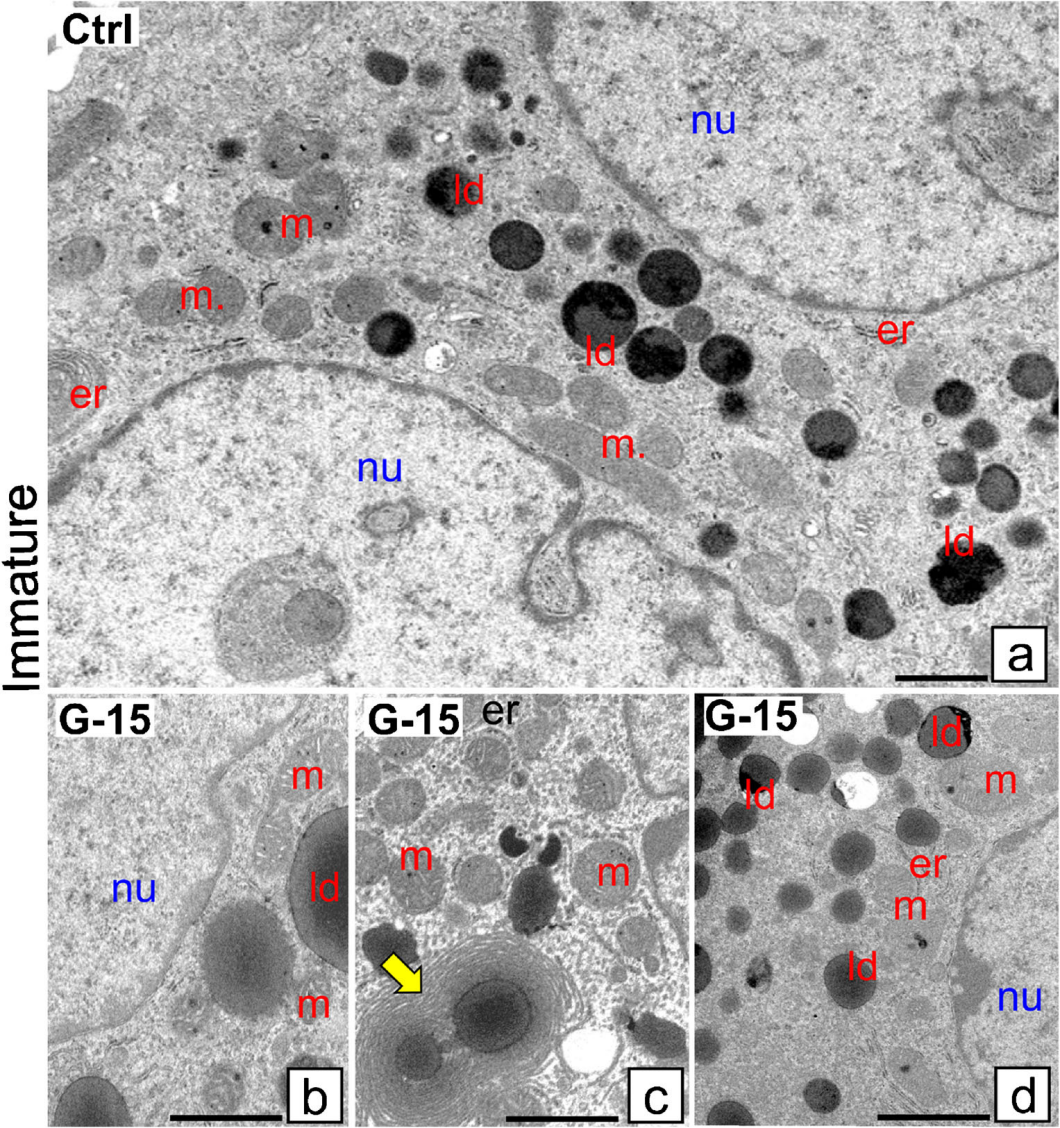
Effect of GPER blockage on Leydig cell ultrastructure. Representative microphotographs of Leydig cells of control and G-15 (50 μg/kg bw)-treated mice. **a**–**d** Immature Leydig cells ultrathin sections. **a** Control immature Leydig cells exhibit normal morphology. In control immature Leydig cells, numerous mitochondria (m) and lipid droplets (ld) are seen (**a**). **b**–**d** After G-15 treatment in immature Leydig cells, more lipid droplets (ld) are observed; some of them are surrounded with concentrically located rough reticulum endoplasmic (er) cisternae (**c**; arrow)

**Fig. 4:**
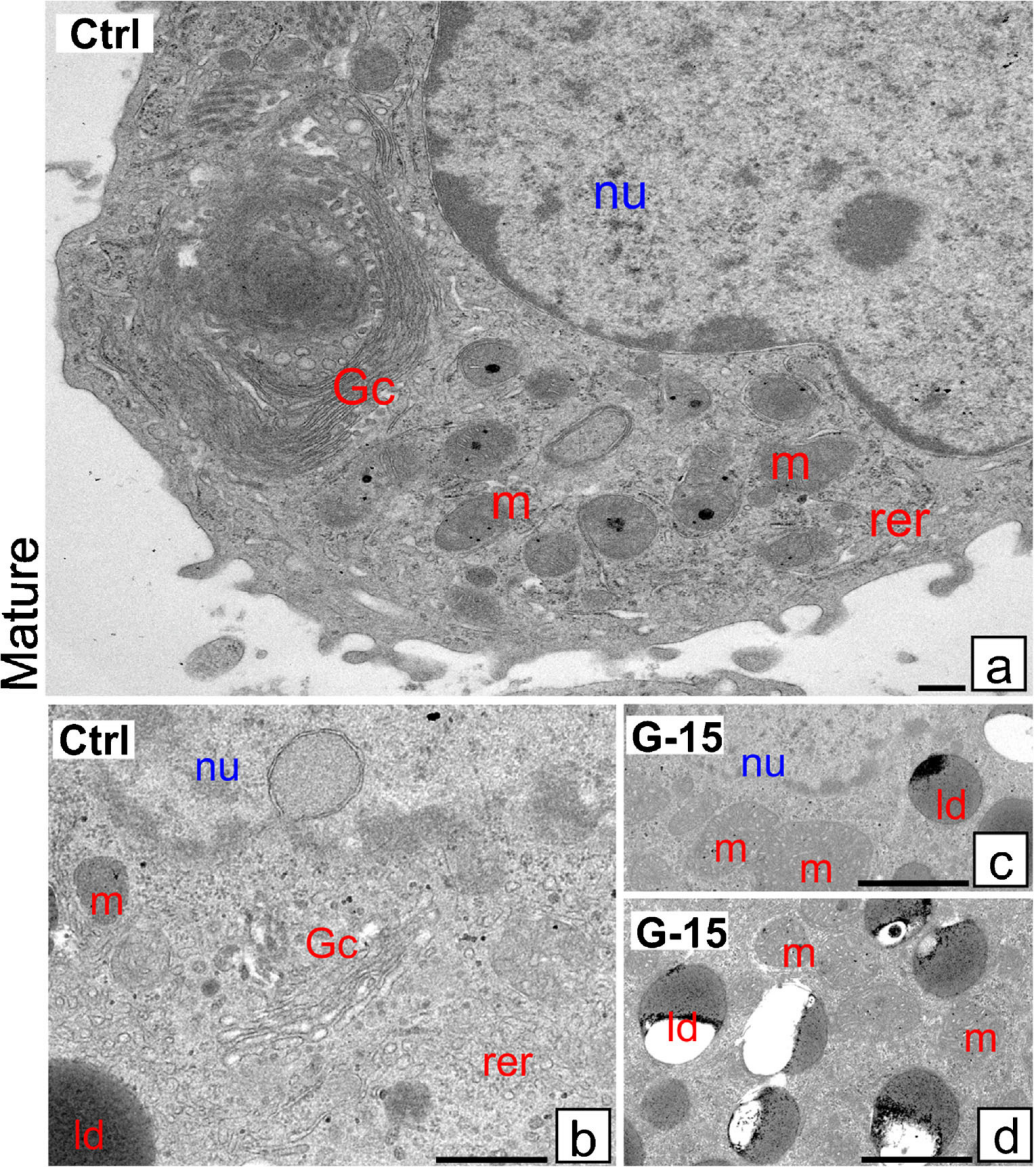
Effect of GPER blockage on Leydig cell ultrastructure. Representative microphotographs of Leydig cells of control and G-15 (50 μg/kg bw)-treated mice. **a**–**d** Mature Leydig cells ultrathin sections. In control mature Leydig cells, lipid droplets (ld) are less numerous. **a**, **b** Mature Leydig cells exhibit normal morphology. Golgi complexes (Gc) and rough ER (rer) are frequently observed (**a**, **b**). **c**, **d** In G-15 mature Leydig cells, large mitochondria (m) and numerous lipid droplets (ld) localized in large accumulations are visible

**Fig. 5:**
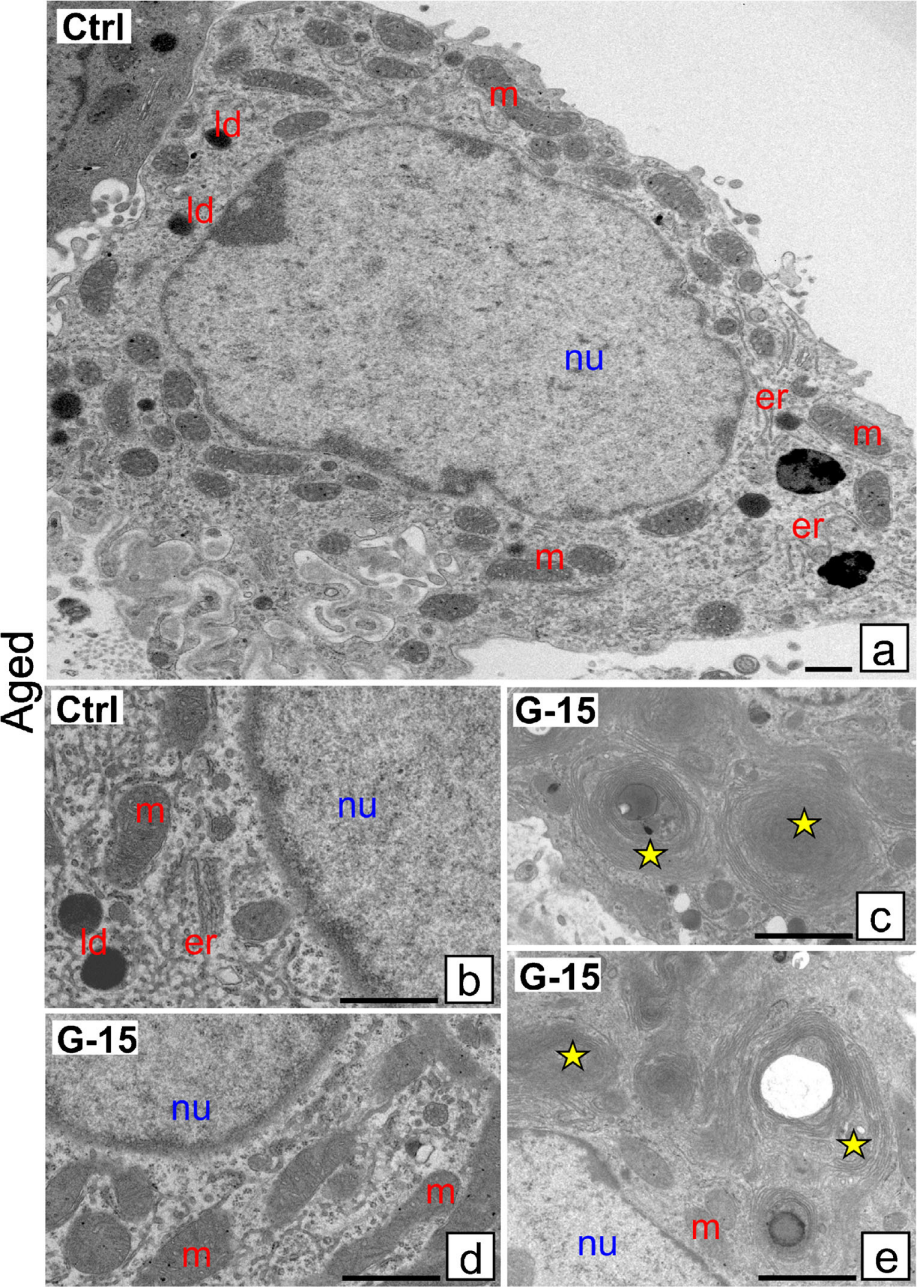
Effect of GPER blockage on Leydig cell ultrastructure. Representative microphotographs of Leydig cells of control and G-15 (50 μg/kg bw)-treated mice. **a**–**e** Aged mouse Leydig cells ultrathin sections. Each testicular sample in the epoxy resin block was cut for at least three ultrathin sections that were analyzed. Bars represent 1 μm. Analysis was performed on testicular blocks from at least three animals of each experimental group. Aged Leydig cells exhibit normal morphology. In control aged Leydig cells, normal number and localization of endoplasmic reticulum (er), mitochondria (m) and lipid droplets (ld) are seen (**a**, **b**). (**c**–**e**) Note, in G-15 aged Leydig cells, the concentric structure of endoplasmic reticulum (er) cisternae (asterisks; **c**, **e**) in between normal-looking and normal-distributed mitochondria (**c**, **d**). (nu) nucleus

Control immature Leydig cells exhibit normal ultrastructure possessing numerous mitochondria (m) and lipid droplets (ld) (Fig. 3a, b). After G-15 treatment more lipid droplets were seen, some surrounded with concentrically located endoplasmic reticulum (er) cisternae, thereby suggesting formation of new lipid droplets (Fig. 3c, d). In control mature Leydig cells, lipid droplets were less numerous than in immature cells (Fig. 4a). Golgi complexes (Gc) and rough endoplasmic reticulum (rer) were frequently seen. In cells treated with G-15, large mitochondria and numerous lipid droplets were revealed (Fig. 4b–d). They were localized in relatively large accumulations, indicating cytoskeleton alternations. In control-aged Leydig cells, the endoplasmic reticulum, mitochondria and lipid droplets were normally distributed (Fig. 5a, b). After G-15 treatment, concentrically in structure endoplasmic reticulum cisternae, probably non-active and degenerating, between normal-looking mitochondria were revealed (Fig. 5c–e).

### Effect of GPER blockage on Leydig cell mitochondrial activity and cytoskeleton structure

After Leydig cell treatment with each of the mentioned agents, no morphological alterations were seen (not shown).

**Fig. 6:**
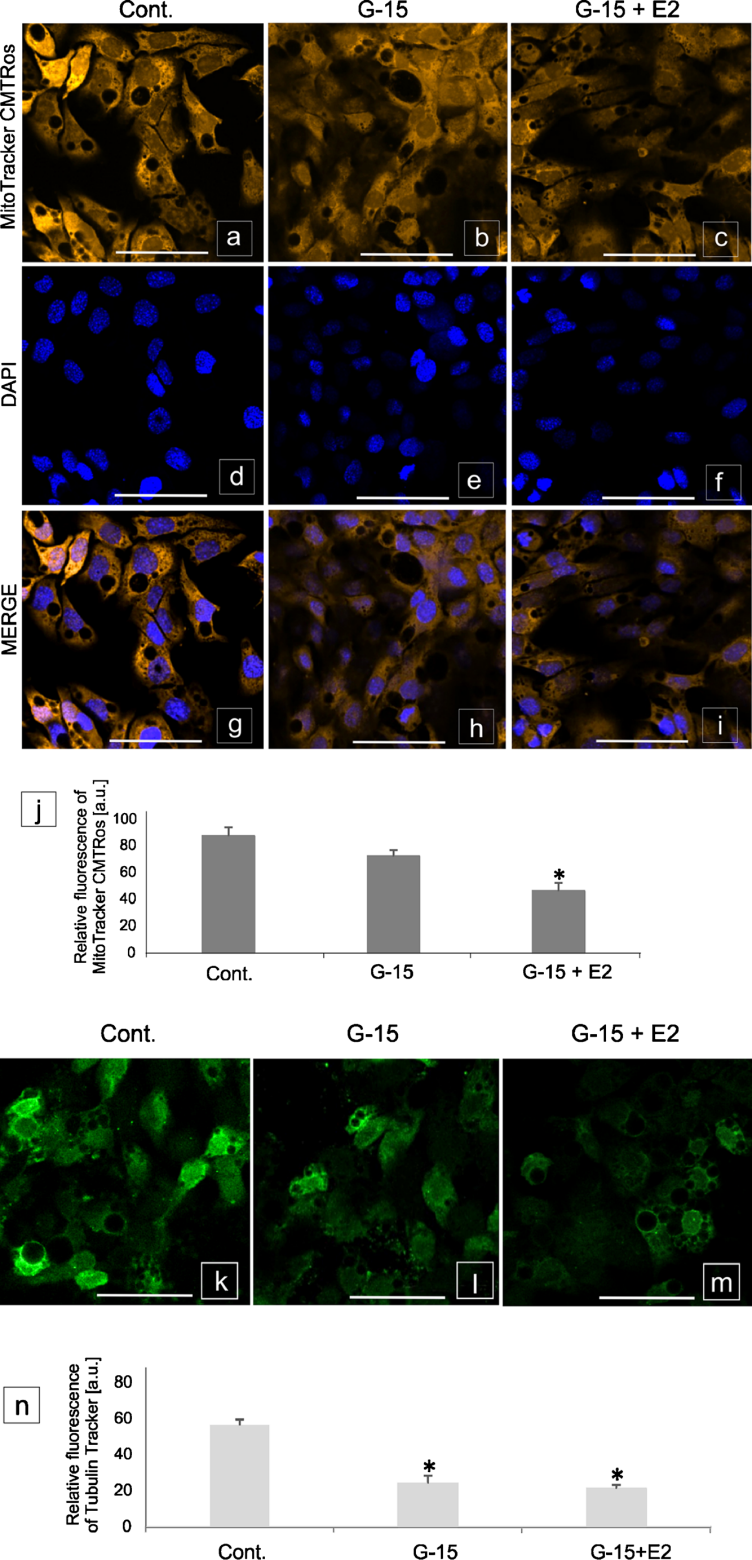
Effect of GPER blockage on Leydig cell mitochondrial activity and cytoskeleton structure. Representative microphotographs and graphs of (**a**–**j**) mitochondrial activity and (**k**–**n**) cytoskeleton structure in control, G-15- and E2- treated MA-10 Leydig cells. Representative microphotographs of cellular localization of MitoTracker (**a**–**i**) in cytoplasm of control (**a**, **g**), G-15 (**b**, **h**) and G-15 with E2 (17β-estradiol) (**c**, **i**)-treated Leydig cells. Immunofluorescence with DAPI (**d**–**f**). Representative microphotographs of cellular localization of TubulinTracker in cytoplasm of control (**k**), G-15 (**l**) and G-15 with E2 (**m**)-treated Leydig cells. Fluorescence without DAPI. Scale bars represent 20 μm. Samples of cultured Leydig cells were measured in triplicate. Quantitative analysis of fluorescence of MitoTracker (**j**) and TubulinTracker (**n**). Histograms of fluorescent intensities expressed as relative fluorescence (arbitrary units; a.u.). Data are expressed as means ± SD. Asterisks show significant differences between control and G-15 (50 μg/kg bw) - treated mouse testes and control and G-15 (10 nM), E2 (10 nM) - treated cells for 24 h. Values are denoted as ^*^ *p* < 0.05

Control Leydig cells were undisturbed, exhibiting high mitochondrial activity (Fig. 6a–i and j) and a normal tubulin cytoskeleton structure (Fig. 6k–m and n). In cells treated with G-15, decrease in mitochondrial (*p* < 0.05) and tubulin activity was revealed. A marked decrease (*p* < 0.05) in mitochondria function and tubulin structure (*p* < 0.05) was found after treatment with G-15 and E2. After treatment with ICI and E2 alone or in combination (ICI + E2 and ICI + G-15), no significant changes in the mitochondria and tubulin activity were revealed (not shown).

### Effect of GPER blockage on mRNA expression of aromatase and estrogen receptors α and β in mouse testes and Leydig cells in vitro

**Fig. 7:**
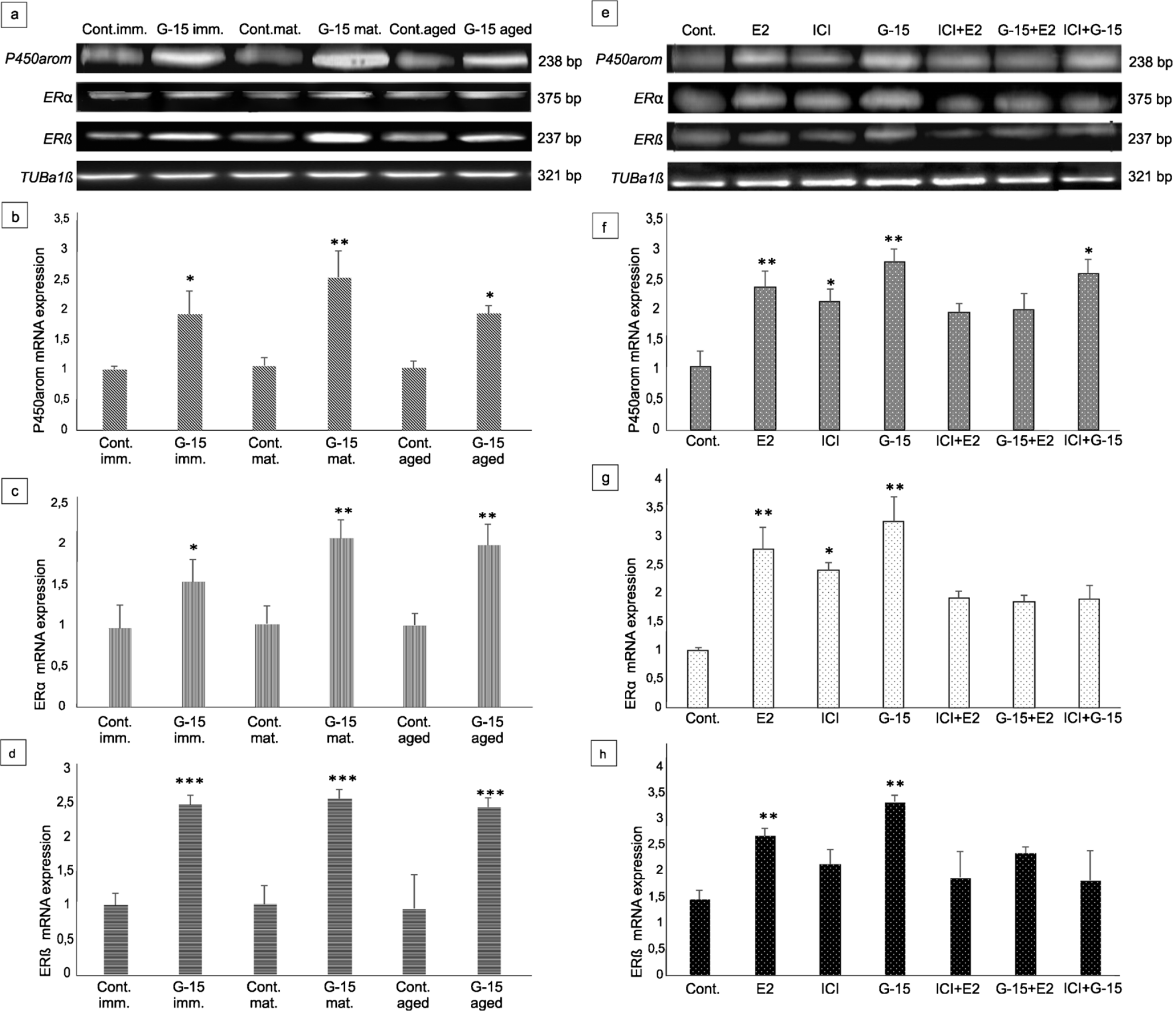
Effect of GPER blockage on mRNA expression of aromatase and estrogen receptors α and β in mouse testes and Leydig cells in vitro*.* (**a**, **e**) Representative gel electrophoresis of qualitative expression of P450aromatase, ERα, ERβ in mouse testes (immature, mature and aged) (**a**) and MA-10 Leydig cells (**e**). (**b**–**d**, **f**–**h**) Relative level (relative quantification; RQ) of mRNA for P450aromatase (**b**), ERα (**c**), ERβ (**d**) in mouse testes (**b**–**d**) and MA-10 Leydig cells (**f**–**h**), determined using real-time RT-PCR analysis 2 − ΔCt method. As an intrinsic control, the tubulin α1a mRNA level was measured in the samples [(**a**, **e**) -qualitative expression]. RQ is expressed as means ± SD. Asterisks show significant differences between control mice and those treated with G-15 (50 μg/kg bw) and control MA-10 Leydig cells and treated with G-15 (10 nM), ICI (ICI 182,780; 10 μM), E2 (17β-estradiol; 10 nM) alone and in combination for 24 h. Values are denoted as ^∗^ *p* < 0.05, ^∗∗^ *p* < 0.01 and ^∗∗∗^ *p* < 0.001. From each animal, at least three samples were measured. Samples of cultured Leydig cells were measured in triplicate

Electrophoresis revealed PCR-amplified products of the predicted sizes: 375 bp for *ERα*, 237 bp for *ERβ*, 238 bp for *P450arom* and 321 bp for *tubulin a1α* (*TUBa1α;* reference gene) in both mouse testes and Leydig cells in vitro (Fig. 7a–h). Real-time RT-PCR analysis in testes of immature, mature and aged mice, both control and experimental (G-15-treated), as well as control and experimental Leydig cells (G-15-, E2-, ICI-treated alone or in combination), revealed changes in the expression level of the studied genes (Fig. 7a–h). No marked differences in *TUBa1α* levels were revealed either in control and experimental mouse testes of various age or MA-10 Leydig cells (Fig. 7a, e).

In treated mice of different ages, a similar trend in expression of estrogen receptors and aromatase was found. In testes with G-15 treatment, significant increase in expressions of ERα (*p* < 0.05; *p* < 0.01), ERβ (*p* < 0.001) and P450arom (*p* < 0.05; *p* < 0.01) were observed when compared to their respective controls (Fig. 7a–d).

In in vitro Leydig cells, the same tendency of in vivo expression of the respective genes was revealed (Fig. 7e–h). Treatment with E2, ICI, or G-15 alone increased the expressions of ERα (*p* < 0.05; *p* < 0.01), ERβ (*p* < 0.01) and P450arom (*p* < 0.05; *p* < 0.01). Treatment of Leydig cells with E2, ICI, or G-15 in combination increased expression of both estrogen receptors and aromatase in comparison to controls; however, the increase was lower (only significant for aromatase *p* < 0.05) when compared to the individual treatments with these agents.

### Effect of GPER blockage on aromatase and estrogen receptors localization in mouse testes and Leydig cells in vitro

**Fig. 8:**
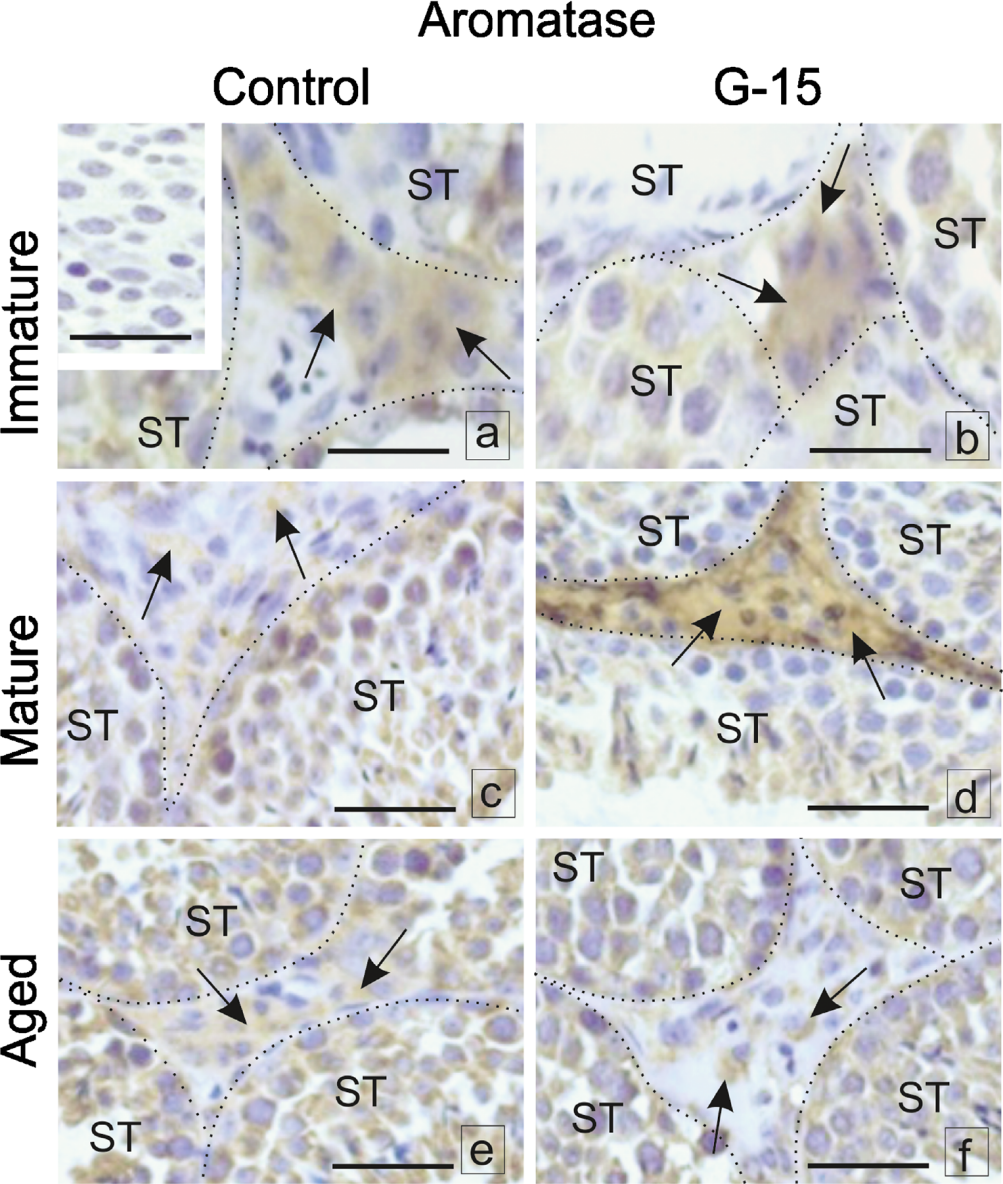
Effect of GPER blockage on aromatase and estrogen receptors localization in mouse testes. Representative microphotographs of testicular sections of control and G-15 (50 μg/kg bw)-treated immature, mature and aged mice. **a**–**f** Localization of P450aromatase; dashed lines (**a**–**f**) mark the periphery of seminiferous tubules (ST). Immunostaining with DAB and counterstaining with hematoxylin. Scale bars represent 15 μm. Immunoreaction was performed on testicular serial sections from at least three animals of each experimental group. No changes in expression of aromatase in testes of immature males after G-15 treatment in comparison to control are seen (arrows) (**a**, **b**). Increase of aromatase expression is visible in Leydig cells of mature G-15 males while its expression is very weak in control ones (arrows) (**c**, **d**). In negative controls, no positive staining is seen (inserts **a**). No differences between expression of aromatase are visible in control and G-15 aged mice (**e**, **f**). In negative controls, no positive staining is seen

**Fig. 9:**
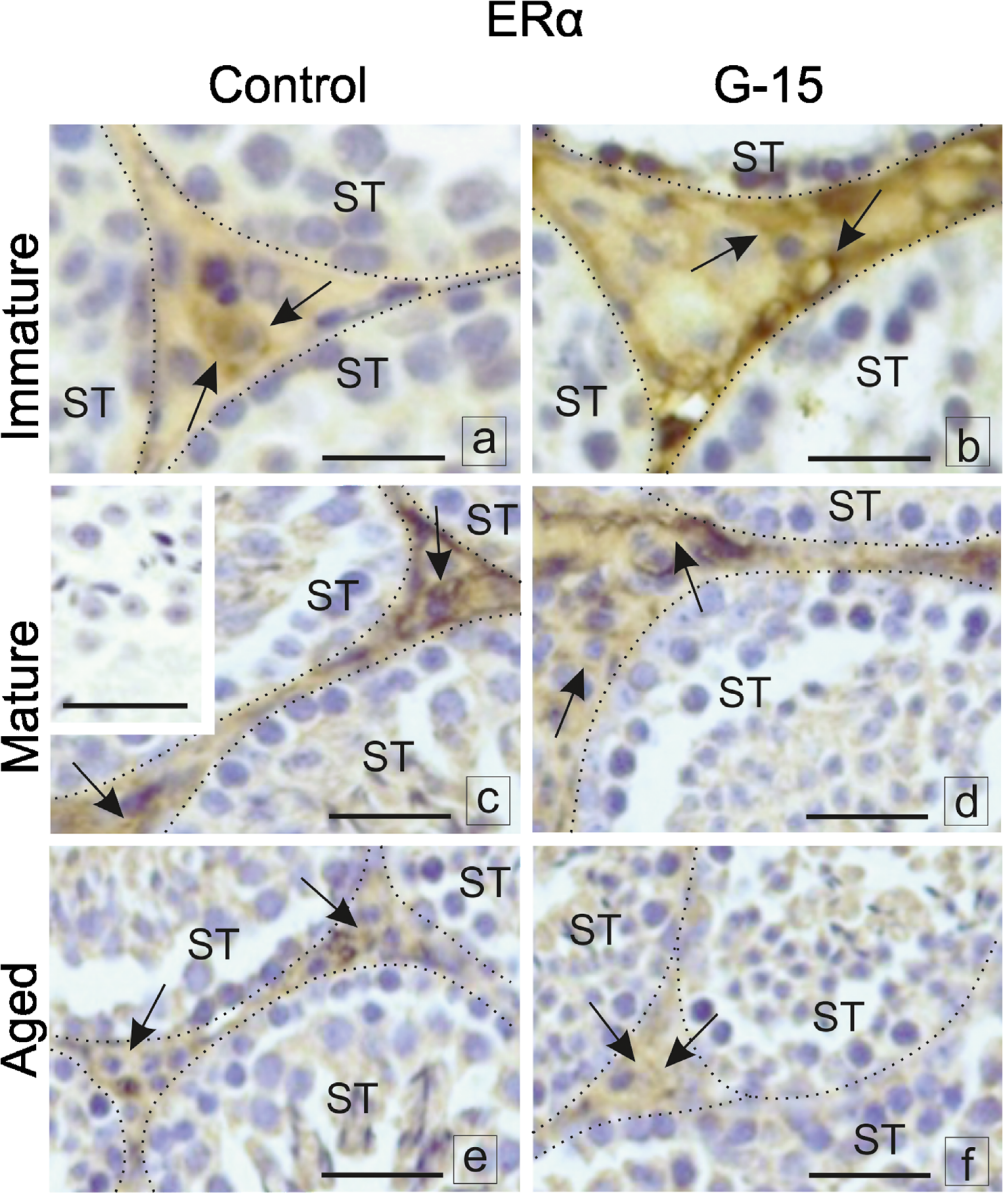
Effect of GPER blockage on aromatase and estrogen receptors localization in mouse testes. Representative microphotographs of testicular sections of control and G-15 (50 μg/kg bw)-treated immature, mature and aged mice. (**a**–**f**) Localization of ERα. Expression of aromatase in both groups is moderate. Slight increase in ERα expression in nuclei and partially in cytoplasm of Leydig cell is observed in G-15 immature mice (arrows) (**a**, **b**). In both mature and aged mice (control and G-15-treated) no changes in expression of ERα is seen (arrows) (**c**–**f**). In negative controls, no positive staining is seen (inserts **c**). The immunoexpression in cytoplasm and nuclei of Leydig cells is strong in mature males while it is moderate in aged ones (arrows) (**c**–**f**)

**Fig. 10:**
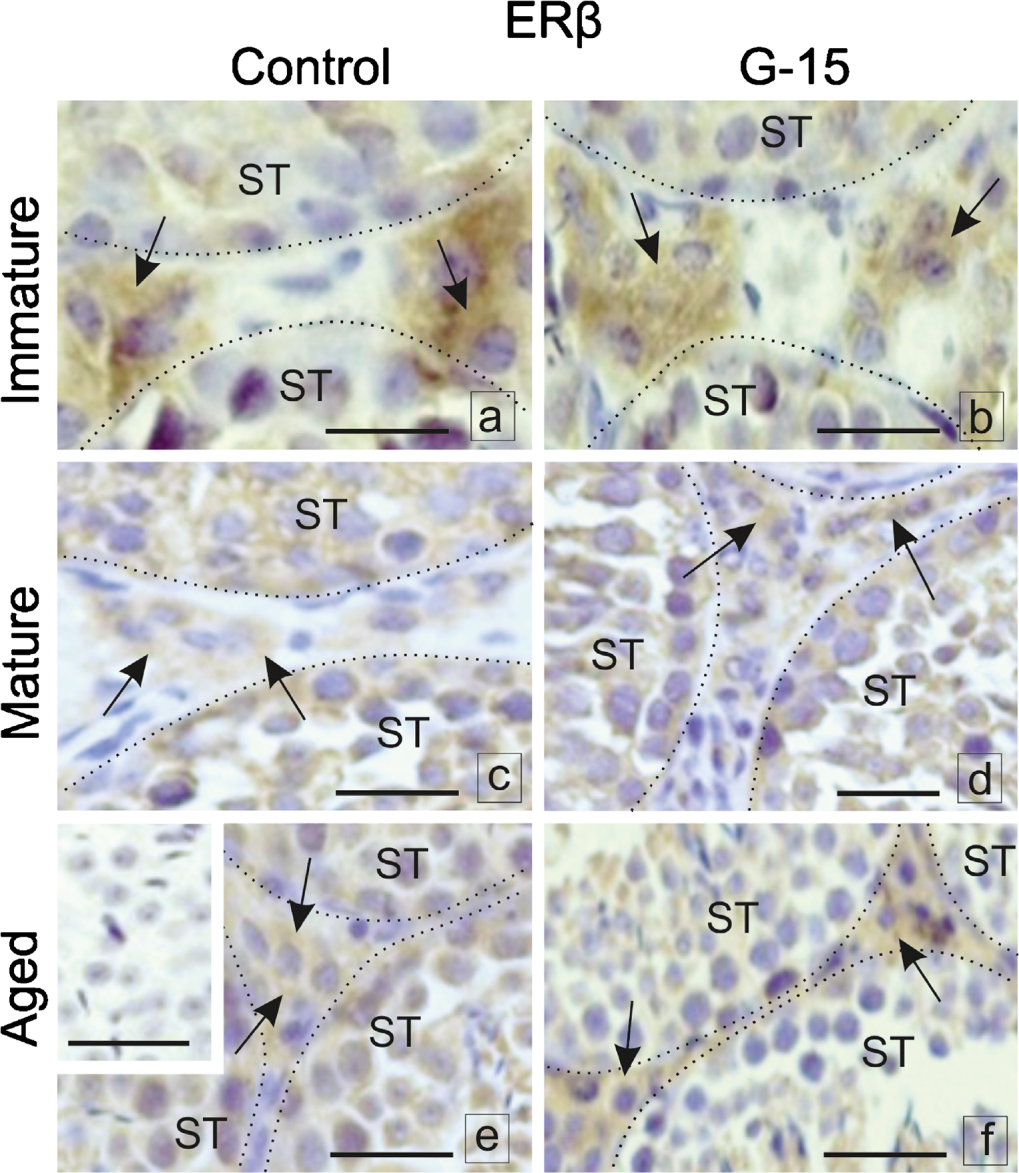
Effect of GPER blockage on aromatase and estrogen receptors localization in mouse testes. Representative microphotographs of testicular sections of control and G-15 (50 μg/kg bw)-treated immature, mature and aged mice. Localization of ERβ (**a**–**f**) in testes of control and G-15-treated mice, respectively. Slight decrease in ERβ expression is observed in cytoplasm of immature males when compared to control whose expression is strong (arrows) (**a**, **b**). Moderate in control mature and weak in G-15 mature males expression of ERβ is revealed exclusively in cytoplasm of Leydig cells (arrows) (**c**, **d**). In control aged males, moderate cytoplasmic expression of ERβ is seen (arrows) (**e**) but is nuclear in a few Leydig cells of G-15 males (arrows) (**f**). In negative controls, no positive staining is seen (inserts **e**). Immunoreaction was performed on testicular serial sections from at least three animals of each experimental group

When blocked by G-15, immunoexpression of aromatase, ERα and ERβ in immature, mature, or aged Leydig cells was altered (Fig. 8a–f). In testes of immature males, no changes in aromatase expression after G-15 treatment were found when compared to controls (Fig. 8a, b). Immunostaining was observed in the cytoplasm of all Leydig cells. Increased aromatase expression was found in Leydig cells of G-15-treated mature males while downregulated expression was observed in control ones (Fig. 8c, d). No difference in expression of aromatase was noted between control and G-15-treated aged mice (Fig. 8e, f). Immunoexpression of aromatase in both groups was of moderate intensity. A slight increase in ERα expression in the nuclei and in Leydig cell cytoplasm was noted after G-15 treatment of immature mice (Fig. 9a, b). In both mature and aged controls and G-15-treated, no change in ERα expression was observed. Increased expression was seen in mature males while moderate expression was observed in the nuclei and cytoplasm of aged Leydig cells (Fig. 9c–f). A slight decrease in ERβ expression was found in the cytoplasm of immature males in comparison to controls (Fig. 10a, b). ERβ expression was moderate in controls and weak in G-15-exposed males and found to be exclusively localized in the cytoplasm of Leydig cells of mature mice (Fig. 10c, d). In control aged males, moderate, cytoplasmic expression of ERβ was found partially localized to the nucleus in Leydig cells of males treated with G-15 (Fig. 10e, f). In negative controls, no positive staining was observed (Figs. 8a, 9c, and 10e).

**Fig. 11:**
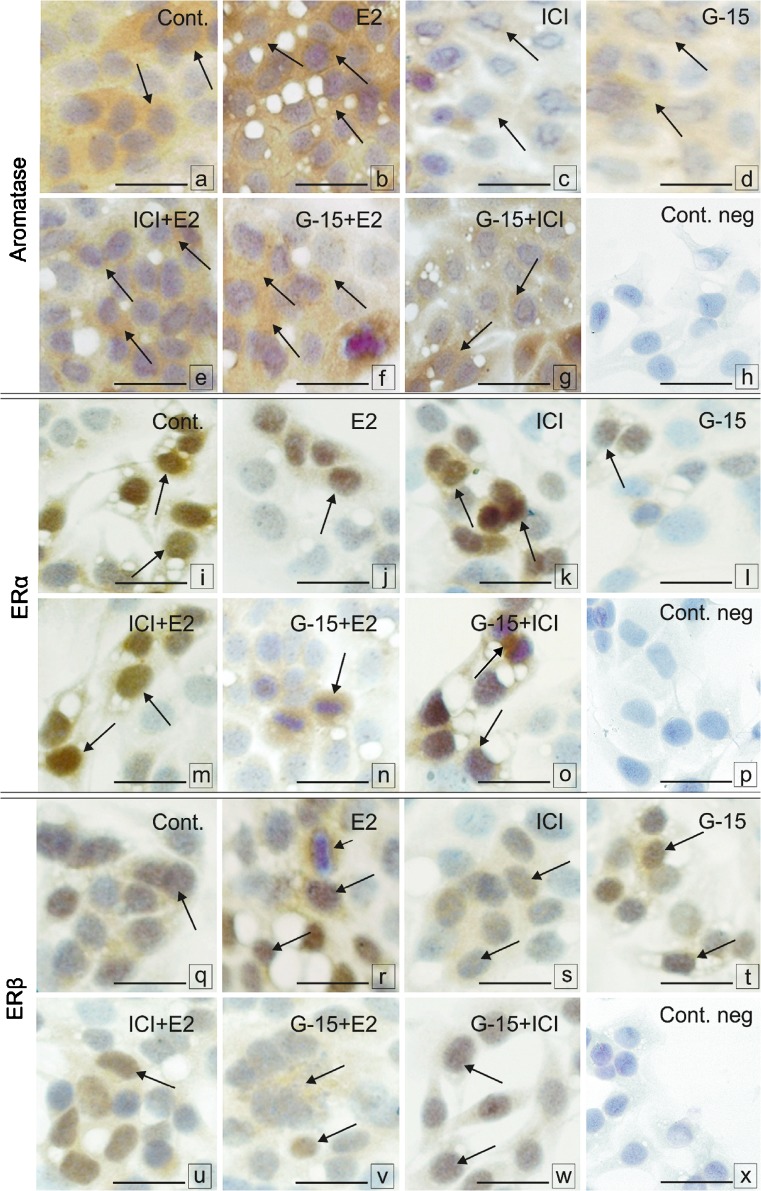
Effect of GPER blockage on localization of aromatase and estrogen receptors in Leydig cells in vitro*.* Representative microphotographs of control MA-10 Leydig cells (**a**, **i**, **q**), E2 (17-β estradiol; 10 nM) (**b**, **j**, **r**), ICI (ICI 182,780; 10 μM) (**c**, **k**, **s**), G-15 (10 nM) (**d**, **l**, **t**), ICI +E2 (**e**, **m**, **u**), G-15+E2 (**f**, **n**, **v**), G-15+ ICI (**g**, **o**, **w**)-treated Leydig cells for 24 h. (**a**–**g**) Localization of P450aromatase; (**i**–**o**) localization of ERα; localization of ERβ (**q**–**w**) in control and G-15-treated Leydig cells. Immunostaining with DAB and counterstaining with hematoxylin. Scale bars represent 20 μm. Cultures of Leydig cells from each experimental group were analyzed in triplicate. Weak to moderate aromatase immunoreaction is seen in the cytoplasm of control Leydig cells (arrows) (**a**). Increase of its expression is visible after E2 treatment (arrows) (**b**). After treatment with ICI weak staining while moderate after G-15 treatment is seen (arrows) (**c**, **d**). After combinations of ICI with E2, G-15 with E2 and G-15 with ICI strong to moderate aromatase expression in a majority of treated cells is seen (arrows) (**e**–**g**). Strong immunoexpression of ERα is observed in nuclei of control Leydig cells (arrows) (**i**). After treatment with both E2 and ICI strong nuclear immunostaining is present in a minority of cells (arrows) (**j**, **k**). After G-15 treatment weak immunostaining in single Leydig cells is visible (arrows), (**l**) which is not the case in cells treated with ICI together with E2 or G-15 (arrows) (**m**, **o**). In cells treated with G-15 together with E2 moderate to strong cytoplasmic immunostaining is seen (arrow) (**n**). Moderate expression of ERβ in a majority of nuclei of control Leydig cells is observed (arrows) (**q**). Increased after E2 treatment immunostaining of ERβ is visible in nuclei of a majority of cells (**r**). In a few cells staining is cytoplasmic (short arrow) (**r**). In a majority of ICI - treated cells the ERβ staining is weak (arrows) (**s**) while after exposure to G-15 and ICI with E2 staining is of strong to moderate intensity and located in nuclei of a majority of cells (arrows) (**t**, **u**). After treatment with G-15 and E2 in combination mainly cytoplasmic staining for ERβ is observed (arrows) (**v**). Moderate to weak nuclear ERβ staining is visible after G-15 and ICI exposure (arrows) (**w**). In negative controls, no positive staining is seen (inserts **h**, **p**, **x**)

Weak to moderate aromatase immunoreaction was found in the cytoplasm of all control Leydig cells (Fig. 11a). Increased aromatase expression was observed after E2 treatment (Fig. 11b). Following treatment with ICI, weak staining was observed while after G-15 moderate staining was revealed (Fig. 11c, d). Combined treatments of ICI with E2, G-15 with E2 and G-15 with ICI resulted in strong to moderate aromatase expression in a majority of treated cells (Fig. 11e–g). Strong immunoexpression of ERα was observed in the nuclei of a majority of control Leydig cells (Fig. 11i). After treatment with both E2 and ICI, strong nuclear immunostaining was present in a minority of cells (Fig. 11j, k). G-15 treatment revealed weak immunostaining in single Leydig cells (Fig. 11l) but that was not seen in cells treated with ICI together with E2 or G-15 (Fig. 11m, n). In cells exposed to G-15 with E2, moderate to strong immunostaining was detected in the cytoplasm (Fig. 11o). Expression of ERβ was moderate in the nuclei of control Leydig cells (Fig. 11q). E2 treatment increased ERβ expression that was still visible in the nuclei, while some cells exhibited cytoplasmic staining (Fig. 11r). In a majority of ICI-treated cells, ERβ staining was weak (Fig. 11s) while, after treatment with G-15 and ICI, E2 staining had strong to moderate intensity and was located in the nuclei of a majority of the cells (Fig. 11t, u). After combined treatment of G-15 and E2, cytoplasmic staining of ERβ was observed (Fig. 11v). Moderate to weak nuclear ERβ staining was detected after G-15 and ICI exposure (Fig. 11w). In negative controls, no positive staining was observed (Fig. 11h, p, and x).

### Effect of GPER on sex steroid concentration in mouse testes and secretion by Leydig cells in vitro

**Fig. 12:**
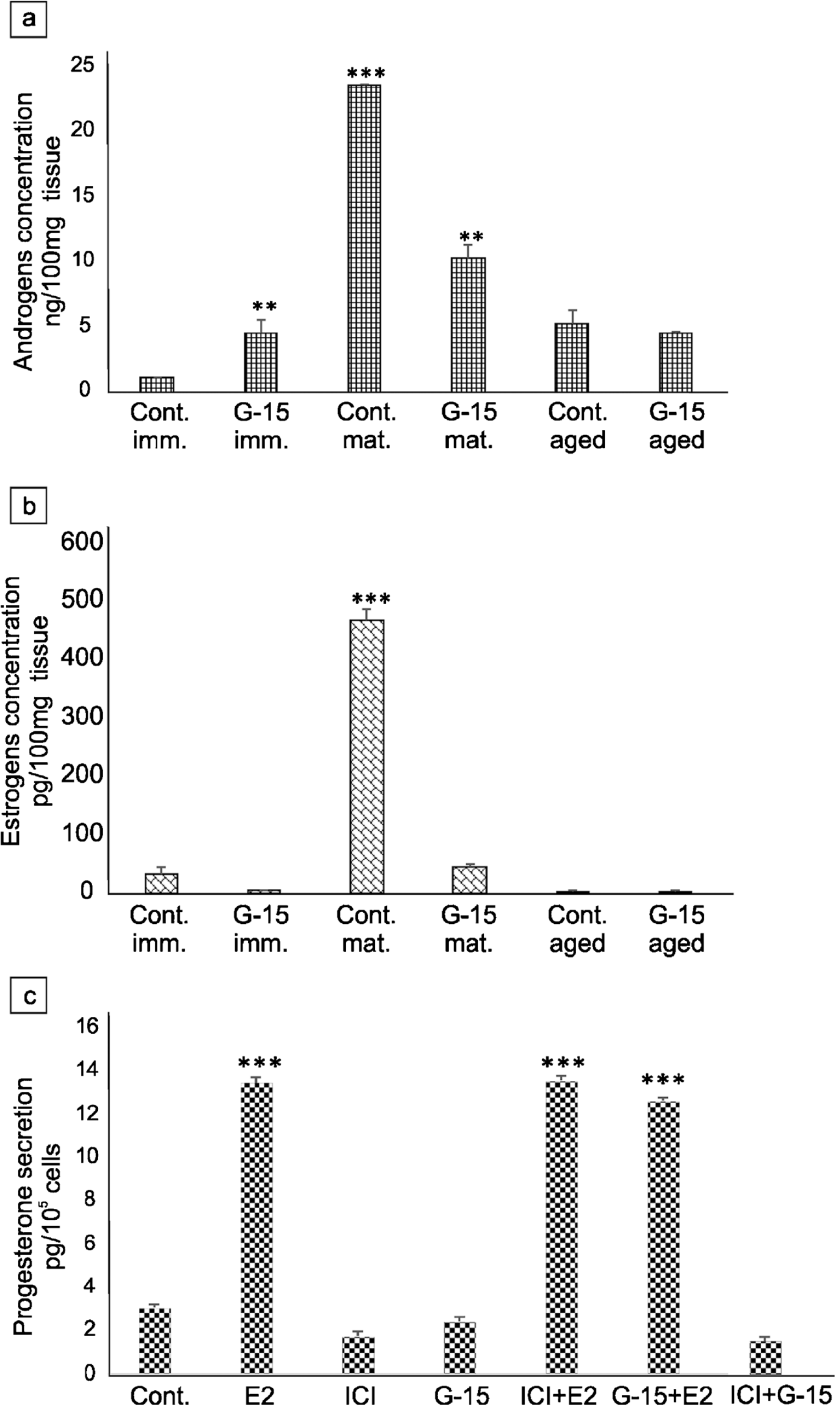
Effect of GPER on sex steroid concentration in mouse testes and secretion by Leydig cells in vitro*.* Androgens and estrogens concentration in testes of immature, mature and aged mice control and G-15-treated (**a**, **b**) and progesterone secretion in MA-10 Leydig cells (**c**) control and G-15, E2 and ICI-treated alone and in combination. Data are expressed as means ± SD. From each animal, at least three samples were measured. Culture media were measured in triplicate. Asterisks show significant differences in testosterone and estradiol concentrations between control and G-15 (50 μg/kg bw)-treated males and in progesterone secretion between control MA-10 Leydig cells and those treated with G-15 (10 nM), ICI (ICI 182,780; 10 μM), E2 (17β-estradiol; 10 nM) alone and in combination for 24 h. Values are denoted as ∗∗*p* < 0.01 and ∗∗∗*p* < 0.001

The highest androgen concentration was revealed in mature mice when compared to immature and aged (Fig. 12a). After treatment with G-15, intratesticular androgen concentration significantly increased (*p* < 0.01) in immature mice while it decreased (*p* < 0.001) in mature males. In both aged controls and G-15-treated animals, no changes in androgen concentrations were found. Intratesticular estrogen concentrations were significantly lower in immature and aged males in comparison to those in mature animals (Fig. 12b). Treatment with G-15 did not create marked changes in estrogen levels. In mature animals, high amounts of estrogen were decreased pronouncedly (*p* < 0.001) after G-15 exposure.

In Leydig cells, progesterone secretion increased markedly (*p* < 0.001) after treatment with E2 alone or in combination with ICI or G-15 in comparison to controls (Fig. 12c). Neither ICI, G-15 alone, nor in combination decreased progesterone secretion when compared to controls.

## Discussion

Estrogens are essential for reproductive tract development, reproductive function and male fertility. Numerous studies have confirmed these hormones and canonical estrogen receptors (ERα, ERβ) are present at every stage of gonad development (Lemmena et al. 1999; Jefferson et al. 2000; Carreau et al. 2003; Lazari et al. 2009). GPER has been identified in a variety of human and rodent estrogen target tissues; thus, its important role in estrogen signaling is currently highlighted (Chimento et al. 2010; Sandner et al. 2014; Heublein et al. 2012; Zarzycka et al. 2016). GPER has been suggested to play a role in multiple systems: skeletal (under sex-depending manner regulation), immune, cardiovascular and renal (Prossnitz and Barton 2011). However, little is known about the role of GPER in both reproductive and nonproductive cell and tissue biology from birth to aging. Interestingly, in frogs, early expression of GPER and aromatase in neuromasts revealed its importance in lateral line system development (Hamilton et al. 2014). Recent, initial studies by Zhang et al. (2015) demonstrated the involvement of GPER in the proper formation and growth of mouse gubernaculum. In the reproductive system, GPER was shown to regulate the proliferative and apoptotic pathways involved in spermatogenesis throughout rat reproductive development (Lucas et al. 2014). In pachytene spermatocytes and round spermatids, GPER activated the epidermal growth factor receptor/extracellular signal-regulated kinases (EGFR/ERK) pathway, thereby inducing the transcriptional modulation of genes controlling apoptosis and differentiation (Chimento et al. 2014).

In steroidogenic Leydig cells, estrogen supervises overall growth, development and function of these cells acting both as a modulator of precursor populations while also inhibiting steroidogenesis via the effect on LH (Abney 1999).

The action of GPER in estrogen signals requires much consideration. The results presented here are the first to report on the influence of GPER on testicular Leydig cell morphology and function. Intratesticular estrogen concentrations, as well as balanced estrogen and androgen concentrations, are crucial for undisturbed testis function. Any change in the sex hormone environment leads to alterations in Leydig cell physiology that affects the action of seminiferous tubules and vice versa. Our results add to the current knowledge that individual cells/tissues may modulate estrogen action via GPER in response to intrinsic factors (e.g., gender, age, genes activity) or extrinsic modulators of estrogen (e.g., synthetic estrogens/environmental estrogens) to determine its function (Filardo and Thomas 2012; Plante et al. 2012). It is not surprising that the highest expression of GPER exists in mature testis as related to a fully developed and functional reproductive system. Herein, histological observations in GPER-blocked testis are in accord with studies in knockout ERβ where increases in Leydig cells per testis correlate with increased steroidogenic capacity (Gould et al. 2007). Based on literature data confirming the proliferative nature of estrogen via both GPER and ER with a contribution of rapid signaling pathways, this effect can be common and/or shared in a large amount by one receptor (Lucas et al. 2011; Chimento et al. 2014; Magruder et al. 2014). On the contrary, in knockout ERα, males’ hypertrophy and loss of Leydig cells with no disturbances of intratesticular steroid levels were demonstrated. Our recent study showed overgrowth of Leydig cells after disturbance of sex hormones milieu, through blockage or activation of estrogen-related receptors (ERRs) in seasonal rodent, the bank vole (Pawlicki et al. 2017). In addition, similar observations were made in testes of boars treated neonatally with antiandrogen (Kotula-Balak et al. 2013). In consequence, aromatase overexpression in overgrown Leydig cells was revealed. In patients with germ cell tumors, elevated levels of chorionic gonadotropin are one possible cause of Leydig cell hypertrophy and hyperplasia (Zimmerli and Hedinger 1991). There are multiple other potential mechanisms where chemicals might induce hyperplasia primarily through a disruption in the hypothalamic-pituitary-axis. In most of these mechanisms, various hormones (e.g., estrogen, prolactin) produce an elevation in LH levels that excessively stimulate steroidogenic Leydig cell function (Greaves 2012).

The use of chemical agents that perturb cytoskeletal protein polarization has shed light on a key role for microfilaments and microtubules in regulating the uptake and transport of cholesterol in steroidogenic cells. Sewer and Li (2008) reported that trafficking of the mitochondria is dependent on microtubules, suggesting cytoskeleton function is necessary in steroid hormone production, particularly at steps subsequent to cholesterol delivery to the mitochondria. Therefore, in steroidogenic cells, proper function of both mitochondria and cytoskeleton strongly guarantees effective sex steroid production. The lack of GPER signaling in mouse testis and Leydig cells affects the action of the above-mentioned cellular structures. In addition, estrogen signaling that is partially disturbed by GPER inactivation modulates the mitochondria and tubulin fiber activity in Leydig cells by estrogen binding to other estrogen receptors and/or via non-receptor estrogen signaling molecules. However, due to changes in gene expression of GPER, ERs and aromatase, such effects are compensational and/or are directed by one molecule. Of note, robust evidence indicates the same nuclear ERs are also located at the cell plasma membrane by palmitoylation and associate with specific membrane proteins, e.g., caveolin 1 (Pietras and Szego 1977; Razandi et al. 1999; Jakacka et al. 2002). Hence, these receptors cannot be also excluded from considerations. It is well-known that different ERα and ERβ isoforms have a very diverse influence on estrogen signaling and target gene regulation (Chang et al. 2008; Vrtačnik et al. 2014). For example, some ERβ isoforms lack the ability to bind ligands or coactivators and certain isoforms affect ERα/ERβ heterodimerization, leading to silenced ERα signaling. It has also been reported that ERβ has an inhibitory effect on ERα-mediated signaling (Murphy 2011). In some instances, opposite roles of ERα and ERβ have been revealed by differences in their expression in various tissues and organs (Girdler and Brotherick 2000; Hess 2003; Roa et al. 2008; Wang et al. 2016). Based on revealed herein GPER, ERs and aromatase genes expression changes, we suggest the existence of an interaction between these genes in mouse Leydig cells of various age. Of note, any change in GPER function results in estrogen signaling modulation, leading to changes in intracellular estrogen levels and its action in Leydig cells. Additionally, ICI affects expression of estrogen signaling molecules. The antiestrogen ICI 182,780 is similar to estradiol in its ability to decrease its receptor expression by approximately 50% (Alarid et al. 1999; Lonard et al. 2000). Decreased expression of ERα but not ERβ, in rat efferent ductules after ICI treatment was demonstrated by Oliveira et al. (2003). Our prior (Hejmej et al. 2011; Kotula-Balak et al. 2013; Zarzycka et al. 2016) and present results confirm that ER expression is regulated by different mechanisms and is dependent on tissue type and species studied. Interestingly, cytoplasmic localization of ERs, coupled with their occasional absence in Leydig cells, can be linked to a dynamic equilibrium between cytoplasm and nucleus. Thus, ERs are not detected at all times in a single subcellular compartment, particularly when the hormonal milieu is changing (Parikh et al. 1987). Neonatal diethylstilbestrol (DES) treatment also affects ERα immunoexpression in the male rat reproductive tract (Rivas et al. 2002).

Estrogen was found to regulate biogenesis and mitochondrial function (Klinge 2008). The localization of ERs in nuclear and ERβ in mitochondrial compartments offers a potential mechanism for controlling coordinate nuclear and mitochondrial gene expression and function of the cell. Bopassa et al. (2010) showed involvement of GPER in the mitochondria permeability transition pore opening in human cardiovascular cells. In human skin fibroblasts, GPER transmitted estrogen signaling through the ERK1/2 pathway that regulated fibroblast cytoskeletal reorganization, leading to changes in cellular shape (Carnesecchi et al. 2015). Moreover, estrogen- and tamoxifen-induced rearrangement of the cytoskeleton and adhesion of the breast cancer cells (MCF-7) (Sapino et al. 1986). In addition, rearrangement of actin and keratin filaments in the cellular projections and the formation of a dense network of keratin fibers took place. The effect was independent of the well-known estrogenic effect on cell proliferation that correlates with a previous study where no proliferation of Leydig cells in vitro and rather volume increase in vivo was observed. Similarly, neither blocked nor activated ERRs induced Leydig cell proliferation (Pawlicki et al. 2017). However, both GPER and ERRs inactivation affected Leydig cell steroidogenic activity by perturbations in various organelle function.

Estradiol controls the intracellular concentration of both ERs in a positive feedback manner. This estrogen-dependent modulation appears to be different for each receptor subtype since estrogen differentially affects the balance between the synthesis and breakdown of ERs (Nilsson et al. 2011; Thomas and Gustafsson 2011). In turn, the presence or absence of a specific ER subtype, as well as its dynamic temporal variations in a cell context co-expressing both receptors (i.e., ERα/ERβ ratio), is pivotal for the appearance of estrogen signaling and dictates the resulting physiological functions. Of note, there is evidence for a role for methylation-dependent modulation of ERβ mRNA and the 26S proteasome in regulating ERβ levels (Pinzone et al. 2004). Estradiol induces ERα phosphorylation that protects the receptor from degradation, indicating how the ERα intracellular concentration is required for the estradiol-evoked cellular effects (Marques et al. 2014). We provided a perspective that addresses mRNA expression changes of estrogen signaling molecules, ERα, ERβ and aromatase in mouse Leydig cells with altered GPER activity. In in vitro Leydig cells, the absence of GPER led to increase of expression of estrogen signaling molecules. Either interaction of ERs with ICI or GPER with G-15 in Leydig cells resulted in comparable and marked estrogen signaling disturbances, indicating the importance of all receptor types and their interactions for controlling steroidognic cell function in the testis. While modulation of ERα and ERβ content is a fundamental factor in estrogen signaling to maintenance of cell number, the lack of GPER and/or changes in expression of ERs and aromatase does not result in cell proliferation or death. Inhibition of Leydig cell regeneration in ethane dimethylsulfonate-treated mature rats following estradiol exposure indicates that Leydig cells self-regulate their number via estrogen modulation in a paracrine fashion (Myers and Abney 1991). Due to these results, involvement of various estrogen receptors in this phenomenon is suggested. Additionally, regulation of intracellular ER and aromatase expression by GPER and vice versa represents a future research direction.

On a new but related point, tamoxifen acting through GPER is able to upregulate aromatase expression (Catalano et al. 2014). In the male reproductive system, GPER suppression of ERα expression and its downstream signaling pathway was reported (Koong and Watson 2014; Jia et al. 2016).

Studies on GPER intracellular localization revealed its presence in the plasma membrane, endoplasmic reticulum and Golgi complex (Chimento et al. 2014). Observed perturbations in these organelles after G-15 treatment are clearly linked. As data are available concerning estrogen ability to activate autophagy, including mitophagy (Lui et al. 2016; Milon et al. 2017), cell context-specific autophagy-based regulation by GPER cannot be excluded. Until today, detailed ultrastructure of GPER deficient cells of various organs has not been performed. The absence of GPER in pancreatic β-cells did not affect cell morphology, although reduced insulin secretion from the pancreas was noted (Sharma and Prossnitz 2011). Our results showed that estrogen signaling molecules control a specific function of various organelles (biogenesis, distribution, and degeneration) in Leydig cells in an age-dependent fashion. The coordinated work of lipid droplets, mitochondria and Golgi apparatus is required for effective steroidogenesis (Cheng and Kowal 1997; Shen et al. 2016). We confirmed that the mitochondria are controlled by estrogens via ERβ and estrogen-related receptors (ERRs) (Giguère 2002; Liao et al. 2015; Milon et al. 2017) and also by GPER along with other estrogen signaling molecules. Recent studies have revealed new proteins associated with lipid droplets, e.g., GPER, where one of the discovered functions in lipid metabolism is upregulation of fatty acids synthesis in cancer cells (Santolla et al. 2012). Data from human embryonic kidney cells (HEK-293) showed that GPER is downregulated through the trans Golgi-proteasome pathway (Cheng et al. 2011), indicating an important GPER role in Golgi complexes function. The presence of GPER in endosomes or intracellular membranes and its capacity to activate plasma membrane receptors, indicates a critical role of GPER in physiological and pathophysiological processes. G protein-coupled receptors (GPCRs) are synthesized in the rough endoplasmic reticulum, traffic through the Golgi apparatus and are dynamically shuttled to and from, the plasma membrane by vesicular transport [e.g., small GTPases, (Rab-1 and Arf) and chaperone transport proteins (COPI/II)] (Martínez-Alonso et al. 2013). GPCRs actions via the activation of plasma membrane-associated enzymes, distinct modes of signal transduction from endosomal membranes, have also been demonstrated (for review see Irannejad and von Zastrow 2014). Surface expression of GPCRs besides extrinsic stimuli can be modulated posttranslationally. Delimitation of GPCRs expression or receptors desensitization (including complete termination of receptor signaling, “downmodulation” by 26S proteasome) affects receptor binding sites and signaling activity (Jean-Alphonse and Hanyaloglu 2011). A major mechanism by which GPCRs promote endosome-initiated signaling is by recruitment of arrestins, which are endocytic adaptors during receptor desensitization and scaffold for the assembly of mitogen-activated protein kinase (MAPK) signaling modules (Shenoy and Lefkowitz 2011).

GPER signaling is a crucial steroidogenesis regulation by estradiol in rat and human LH-stimulated Leydig cells. The detrimental effects of estrogen excess after estradiol or GPER agonist treatment on steroidogenesis were revealed (Hess 2003; Vaucher et al. 2014). Similarly, in fish gonads, the GPER effect on steroidogenesis has been shown (Thomas et al. 2006; Pang and Thomas 2010). Therefore, decreased estrogen concentration in mice without GPER (and modulated expression of ERs and aromatase genes) confirmed a full set of estrogen signaling molecule control of Leydig cell steroidogenic function at the ultrastructure (organelle activity) and molecular (hormone production and secreting ability) levels.

## Conclusion

The GPER provides a new basis for understanding the roles of estrogen and estrogen signaling molecules in the regulation of Leydig cells function. The data presented here revealed that GPER has a regulatory effect on estrogen signaling, as well as estrogen concentration and secretion in mouse Leydig cells (dependent of male age and putative engagement of various organelle) via its partnership with ERs and aromatase. Thus, the generation of a triple KO murine model (aromatase, ER, and GPER) could be an important contribution to further understand the mechanisms of estrogen signaling in Leydig cells.

## References

[CR1] Abney TO (1999). The potential roles of estrogens in regulating Leydig cell development and function: a review. Steroids.

[CR2] Abney TO, Myers RB (1991). 17 beta-estradiol inhibition of Leydig cell regeneration in the ethane dimethylsulfonate-treated mature rat. J Androl.

[CR3] Abraham GE, Swerdloff ES, Tulchinsky D, Odell WD (1971). Radioimmunoassay of plasma 17-hydroxyprogesterone. J Clin Endocrinol.

[CR4] Acconcia Filippo, Fiocchetti Marco, Marino Maria (2017). Xenoestrogen regulation of ERα/ERβ balance in hormone-associated cancers. Molecular and Cellular Endocrinology.

[CR5] Alarid ET, Bakopoulos N, Solodin N (1999). Proteasome-mediated proteolysis of estrogen receptor: a novel component in autologous down-regulation. Mol Endocrinol.

[CR6] An G, Li W, Yan T, Li S (2014). Estrogen rapidly enhances incisional pain of ovariectomized rats primarily through the G protein-coupled estrogen receptor. Int J Mol Sci.

[CR7] Antal MC, Krust A, Chambon P, Mark M (2008). Sterility and absence of histopathological defects in nonreproductive organs of a mouse ERbeta-null mutant. Proc Natl Acad Sci U S A.

[CR8] Ascoli M (1981). Characterization of several clonal lines of cultured Leydig tumor cells: gonadotropin receptors and steroidogenic responses. Endocrinology.

[CR9] Atanassova N, McKinnell C, Walker M, Turner KJ, Fisher JS, Morley M, Millar MR, Groome NP, Sharpe RM (1999). Permanent effects of neonatal estrogen exposure in rats on reproductive hormone levels, Sertoli cell number, and the efficiency of spermatogenesis in adulthood. Endocrinology.

[CR10] Barakat R, Oakley O, Kim H, Jin J, Myong C, Ko J (2016). Extra-gonadal sites of estrogen biosynthesis and function. BMB Rep.

[CR11] Bertrand S, Hu C, Aksenova MV, Mactutus CF, Booze RM (2015). HIV-1 Tat and cocaine mediated synaptopathy in cortical and midbrain neurons is prevented by the isoflavone Equol. Front Microbiol.

[CR12] Bopassa JC, Eghbali M, Toro L, Stefani E (2010). A novel estrogen receptor GPER inhibits mitochondria permeability transition pore opening and protects the heart against ischemia-reperfusion injury. Am J Physiol Heart Circ Physiol.

[CR13] Carnesecchi J, Malbouyres M, de Mets R, Balland M, Beauchef G, Vié K, Chamot C, Lionnet C, Ruggiero F, Vanacker JM (2015). Estrogens induce rapid cytoskeleton re-organization in human dermal fibroblasts via the non-classical receptor GPR30. PLoS One.

[CR14] Carreau S, Hess RA (2010). Oestrogens and spermatogenesis. Philos Trans R Soc Lond Ser B Biol Sci.

[CR15] Carreau S, Lambard S, Delalande C, Denis-Galeraud I, Bilinska B, Bourguiba S (2003). Aromatase expression and role of estrogens in male gonad : a review. Reprod Biol Endocrinol.

[CR16] Carreau S, Bouraima-Lelong H, Delalande C (2012). Role of estrogens in spermatogenesis. Front Biosci (Elite Ed).

[CR17] Catalano S, Giordano C, Panza S, Chemi F, Bonofiglio D, Lanzino M, Rizza P, Romeo F, Fuqua SA, Maggiolini M, Andò S, Barone I (2014). Tamoxifen through GPER upregulates aromatase expression: a novel mechanism sustaining tamoxifen-resistant breast cancer cell growth. Breast Cancer Res Treat.

[CR18] Chang EC, Charn TH, Park S-H, Helferich WG, Komm B, Katzenellenbogen JA, Katzenellenbogen BS (2008). Estrogen receptors α and β as determinants of gene expression: influence of ligand, dose, and chromatin binding. Mol Endocrinol.

[CR19] Cheng B, Kowal J (1997). Role of the Golgi complex in adrenocortical steroidogenesis. Microsc Res Tech.

[CR20] Cheng SB, Quinn JA, Graeber CT, Filardo EJ (2011). Down-modulation of the G-protein-coupled estrogen receptor, GPER, from the cell surface occurs via a trans-Golgi-proteasome pathway. J Biol Chem.

[CR21] Chimento A, Sirianni R, Delalande C, Silandre D, Bois C, Ando S, Maggiolini M, Carreau S, Pezzi V (2010). 17 beta-estradiol activates rapid signaling pathways involved in rat pachytene spermatocytes apoptosis through GPR30 and ER alpha. Mol Cell Endocrinol.

[CR22] Chimento A, Sirianni R, Zolea F, Bois C, Delalande C, Ando S, Maggiolini M, Aquila S, Carreau S, Gper PV (2011). ESRs are expressed in rat round spermatids and mediate oestrogen-dependent rapid pathways modulating expression of cyclin B1 and Bax. Int J Androl.

[CR23] Chimento A, Sirianni R, Casaburi I, Pezzi V (2014). GPER signaling in spermatogenesis and testicular tumors. Front Endocrinol (Lausanne).

[CR24] Couse JF, Korach KS (1999). Estrogen receptor null mice: what have we learned and where will they lead us?. Endocr Rev.

[CR25] Dennis MK, Burai R, Ramesh C, Petrie WK, Alcon SN, Nayak TK, Bologa CG, Leitao A, Brailoiu E, Deliu E, Dun NJ, Sklar LA, Hathaway HJ, Arterburn JB, Oprea TI, Prossnitz ER (2009). In vivo effects of a GPR30 antagonist. Nat Chem Biol.

[CR26] Dennis MK, Field AS, Burai R, Ramesh C, Petrie WK, Bologa CG, Oprea TI, Yamaguchi Y, Hayashi S, Sklar LA, Hathaway HJ, Arterburn JB, Prossnitz ER (2011). Identification of a GPER/GPR30 antagonist with improved estrogen receptor counterselectivity. J Steroid Biochem Mol Biol.

[CR27] Dufau ML, Catt KJ, Tsuruhara T, Ryan D (1972). Radioimmunoassay of plasma testosterone. Clin Chim Acta.

[CR28] Fietz D, Ratzenböck C, Hartmann K, Raabe O, Kliesch S, Weidner W, Klug J, Bergmann M (2014). Expression pattern of estrogen receptors α and β and G-protein-coupled estrogen receptor 1 in the human testis. Histochem Cell Biol.

[CR29] Fietz D, Bergmann M, Hartmann K (2016). In situ hybridization of estrogen receptors α and β and GPER in the human testis. Methods Mol Biol.

[CR30] Filardo EJ, Thomas P (2012). Minireview: G protein-coupled estrogen receptor-1, GPER-1: its mechanism of action and role in female reproductive cancer, renal and vascular physiology. Endocrinology.

[CR31] Gaudet HM, Cheng SB, Christensen EM, Filardo EJ (2015). The G-protein coupled estrogen receptor, GPER: the inside and inside-out story. Mol Cell Endocrinol.

[CR32] Giguère V (2002). To ERR in the estrogen pathway. Trends Endocrinol Metab.

[CR33] Girdler F, Brotherick I (2000). The oestrogen receptors (ER alpha and ER beta) and their role in breast cancer: a review. Breast.

[CR34] Gould ML, Hurst PR, Nicholson HD (2007). The effects of oestrogen receptors alpha and beta on testicular cell number and steroidogenesis in mice. Reproduction.

[CR35] Greaves P (2012). Histopathology of preclinical toxicity studies: interpretation and relevance in drug safety evaluation.

[CR36] Hamilton CK, Navarro-Martin L, Neufeld M, Basak A, Trudeau VL (2014). Early expression of aromatase and the membrane estrogen receptor GPER in neuromasts reveals a role for estrogens in the development of the frog lateral line system. Gen Comp Endocrinol.

[CR37] Hejmej A, Kotula-Balak M, Galas J, Bilińska B (2011). Effects of 4-tert-octylphenol on the testes and seminal vesicles in adult male bank voles. Reprod Toxicol.

[CR38] Hess RA (2000). Oestrogen in fluid transport in efferent ducts of the male reproductive tract. Rev Reprod.

[CR39] Hess RA (2003). Estrogen in the adult male reproductive tract: a review. Reprod Biol Endocrinol.

[CR40] Heublein S, Lenhard M, Vrekoussis T, Schoepfer J, Kuhn C, Friese K, Makrigiannakis A, Mayr D, Jeschke U (2012). The G-protein-coupled estrogen receptor (GPER) is expressed in normal human ovaries and is upregulated in ovarian endometriosis and pelvic inflammatory disease involving the ovary. Reprod Sci.

[CR41] Hoffmann B, Rostalski A, Mutembei HM, Goericke-Pesch S (2010). Testicular steroid hormone secretion in the boar and expression of testicular and epididymal steroid sulphatase and estrogen sulphotransferase activity. Exp Clin Endocrinol Diabetes.

[CR42] Hotchkiss J, Atkinson LE, Knobil E (1971). Time course of serum estrogen and luteinizing hormone concentrations during menstrual cycle of rhesus monkey. Endocrinology.

[CR43] Irannejad R, von Zastrow M (2014). GPCR signaling along the endocytic pathway. Curr Opin Cell Biol.

[CR44] Irsik DL, Carmines PK, Lane PH (2013). Classical estrogen receptors and ERα splice variants in the mouse. PLoS One.

[CR45] Isensee J, Meoli L, Zazzu V, Nabzdyk C, Witt H, Soewarto D, Effertz K, Fuchs H, Gailus-Durner V, Busch D, Adler T, de Angelis MH, Irgang M, Otto C, Noppinger PR (2009). Expression pattern of G protein-coupled receptor 30 in LacZ reporter mice. Endocrinology.

[CR46] Jakacka M, Ito M, Martinson F, Ishikawa T, Lee EJ, Jameson JL (2002). An estrogen receptor (ER)alpha deoxyribonucleic acid-binding domain knock-in mutation provides evidence for nonclassical ER pathway signaling in vivo. Mol Endocrinol.

[CR47] Jean-Alphonse F, Hanyaloglu AC (2011). Regulation of GPCR signal networks via membrane trafficking. Mol Cell Endocrinol.

[CR48] Jefferson WN, Couse JF, Banks EP, Korach KS, Newbold RR (2000). Expression of estrogen receptor beta is developmentally regulated in reproductive tissues of male and female mice. Biol Reprod.

[CR49] Jia B, Gao Y, Li M, Shi J, Peng Y, Du X, Klocker H, Sampson N, Shen Y, Liu M, Zhang J (2016). GPR30 promotes prostate stromal cell activation via suppression of ERα expression and its downstream signaling pathway. Endocrinology.

[CR50] Kang W.-B., Cong Y., Ru J.-Y., Ying S.-Q., Zhu T., Wang D.-S., Liu X.-W., Liu G., Zhao J.-N. (2015). Osteoprotective effect of combination therapy of low-dose oestradiol with G15, a specific antagonist of GPR30/GPER in ovariectomy-induced osteoporotic rats. Bioscience Reports.

[CR51] Klinge CM (2008). Estrogenic control of mitochondrial function and biogenesis. J Cell Biochem.

[CR52] Koong LY, Watson CS (2014). Direct estradiol and diethylstilbestrol actions on early- versus late-stage prostate cancer cells. Prostate.

[CR53] Kotula-Balak M, Hejmej A, Kopera I, Lydka M, Bilinska B (2012). Prenatal and neonatal exposure to flutamide affects function of Leydig cells in adult boar. Domest Anim Endocrinol.

[CR54] Kotula-Balak M, Chojnacka K, Hejmej A, Galas J, Satola M, Bilinska B (2013). Does 4-tert-octylphenol affect estrogen signaling pathways in bank vole Leydig cells and tumor mouse Leydig cells in vitro?. Reprod Toxicol.

[CR55] Krege JH, Hodgin JB, Couse JF, Enmark E, Warner M, Mahler JF, Sar M, Korach KS, Gustafsson JA, Smithies O (1998). Generation and reproductive phenotypes of mice lacking estrogen receptor beta. Proc Natl Acad Sci U S A.

[CR56] Lazari MF, Lucas TF, Yasuhara F, Gomes GR, Siu ER, Royer C, Fernandes SA, Porto CS (2009). Estrogen receptors and function in the male reproductive system. Arq Bras Endocrinol Metabol.

[CR57] Lee KH, Park JH, Bunick D, Lubahn DB, Bahr JM (2009). Morphological comparison of the testis and efferent ductules between wild-type and estrogen receptor alpha knockout mice during postnatal development. J Anat.

[CR58] Lemmena JG, Broekhof JLM, Kuiper GJM, Gustafsson J-A, van der Saag PT, van der Burg B (1999). Expression of estrogen receptor alpha and beta during mouse embryogenesis. Mech Dev.

[CR59] Li X, Li H, Jia L, Li X, Rahman N (2015). Oestrogen action and male fertility: experimental and clinical findings. Cell Mol Life Sci.

[CR60] Liao TL, Tzeng CR, Yu CL, Wang YP, Kao SH (2015). Estrogen receptor-β in mitochondria: implications for mitochondrial bioenergetics and tumorigenesis. Ann N Y Acad Sci.

[CR61] Livak KJ, Schmittgen TD (2001). Analysis of relative gene expression data using real-time quantitative PCR and the 2(-Delta Delta C(T)) method. Methods.

[CR62] Lonard DM, Nawaz Z, Smith CL, O'Malley BW (2000). The 26S proteasome is required for estrogen receptor-alpha and coactivator turnover and for efficient estrogen receptor-alpha transactivation. Mol Cell.

[CR63] Lucas TF, Royer C, Siu ER, Lazari MF, Porto CS (2010). Expression and signaling of G protein-coupled estrogen receptor 1 (GPER) in rat Sertoli cells. Biol Reprod.

[CR64] Lucas TF, Pimenta MT, Pisolato R, Lazari MF, Porto CS (2011). 17Beta-estradiol signaling and regulation of Sertoli cell function. Spermatogenesis.

[CR65] Lucas TF, Nascimento AR, Pisolato R, Pimenta MT, Lazari MF, Porto CS (2014). Receptors and signaling pathways involved in proliferation and differentiation of Sertoli cells. Spermatogenesis.

[CR66] Lui A, New J, Ogony J, Thomas S, Lewis-Wambi J (2016). Everolimus downregulates estrogen receptor and induces autophagy in aromatase inhibitor-resistant breast cancer cells. BMC Cancer.

[CR67] Magruder HT, Quinn JA, Schwartzbauer JE, Reichner J, Huang A, Filardo EJ (2014). G protein-coupled estrogen receptor-1, GPER-1, promotes fibrillogenesis via a shc-dependent pathway resulting in anchorage-independent growth. Horm Cancer.

[CR68] Marques M, Laflamme L, Benassou I, Cissokho C, Guillemette B, Gaudreau L (2014). Low levels of 3,3′-diindolylmethane activate estrogen receptor α and induce proliferation of breast cancer cells in the absence of estradiol. BMC Cancer.

[CR69] Mårtensson UE, Salehi SA, Windahl S, Gomez MF, Swärd K, Daszkiewicz-Nilsson J, Wendt A, Andersson N, Hellstrand P, Grände PO, Owman C, Rosen CJ, Adamo ML, Lundquist I, Rorsman P, Nilsson BO, Ohlsson C, Olde B, Leeb-Lundberg LM (2009). Deletion of the G protein-coupled receptor 30 impairs glucose tolerance, reduces bone growth, increases blood pressure, and eliminates estradiol-stimulated insulin release in female mice. Endocrinology.

[CR70] Martínez-Alonso E, Tomás M, Martínez-Menárguez JA (2013). Golgi tubules: their structure, formation and role in intra-Golgi transport. Histochem Cell Biol.

[CR71] Milon Agnieszka, Opydo-Chanek Malgorzata, Tworzydlo Waclaw, Galas Jerzy, Pardyak Laura, Kaminska Alicja, Ptak Anna, Kotula-Balak Malgorzata (2017). Chlorinated biphenyls effect on estrogen-related receptor expression, steroid secretion, mitochondria ultrastructure but not on mitochondrial membrane potential in Leydig cells. Cell and Tissue Research.

[CR72] Murphy E (2011). Estrogen signaling and cardiovascular disease. Circ Res.

[CR73] Myers RB, Abney TO (1991). Interstitial cell proliferation in the testis of the ethylene dimethane sulfonate-treated rat. Steroids.

[CR74] Nilsson S, Koehler KF, Gustafsson JA (2011). Development of subtype-selective oestrogen receptor-based therapeutics. Nat Rev Drug Discov.

[CR75] Oliveira CA, Nie R, Carnes K, Franca LR, Prins GS, Saunders PT, Hess RA (2003). The antiestrogen ICI 182,780 decreases the expression of estrogen receptor-alpha but has no effect on estrogen receptor-beta and androgen receptor in rat efferent ductules. Reprod Biol Endocrinol.

[CR76] Otto C, Fuchs I, Kauselmann G, Kern H, Zevnik B, Andreasen P, Schwarz G, Altmann H, Klewer M, Schoor M, Vonk R, Fritzemeier KH (2009). GPR30 does not mediate estrogenic responses in reproductive organs in mice. Biol Reprod.

[CR77] Pang Y, Thomas P (2010). Role of G protein-coupled estrogen receptor 1, GPER, in inhibition of oocyte maturation by endogenous estrogens in zebrafish. Dev Biol.

[CR78] Pardyak L, Kaminska A, Galas J, Ptak A, Bilinska B, Kotula-Balak M (2016). Primary and tumor mouse Leydig cells exposed to polychlorinated naphthalenes mixture: effect on estrogen related-receptors expression, intracellular calcium level and sex hormones secretion. Tissue Cell.

[CR79] Parikh I, Rajendran KG, Su JL, Lopez T, Sar M (1987). Are estrogen receptors cytoplasmic or nuclear? Some immunocytochemical and biochemical studies. J Steroid Biochem.

[CR80] Pawlicki P, Milon A, Zarzycka M, Galas J, Tworzydlo W, Kaminska A, Pardyak L, Lesniak K, Pacwa A, Bilinska B, Gorowska-Wojtowicz E, Kotula-Balak M (2017). Does signaling of estrogen-related receptors affect structure and function of bank vole Leydig cells?. J Physiol Pharmacol.

[CR81] Pfaffl MW (2001). A new mathematical model for relative quantification in real-time RT-PCR. Nucleic Acids Res.

[CR82] Pietras RJ, Szego CM (1977). Specific binding sites for oestrogen at the outer surfaces of isolated endometrial cells. Nature.

[CR83] Pinzone JJ, Stevenson H, Strobl JS, Berg PE (2004). Molecular and cellular determinants of estrogen receptor α expression. Mol Cell Biol.

[CR84] Plante BJ, Lessey BA, Taylor RN, Wang W, Bagchi MK, Yuan L, Scotchie J, Fritz MA, Young SL (2012). G protein-coupled estrogen receptor (GPER) expression in normal and abnormal endometrium. Reprod Sci.

[CR85] Prossnitz ER, Barton M (2011). The G protein-coupled estrogen receptor GPER in health and disease. Nat Rev Endocrinol.

[CR86] Razandi M, Pedram A, Greene GL, Levin ER (1999). Cell membrane and nuclear estrogen receptors (ERs) originate from a single transcript: studies of ERalpha and ERbeta expressed in Chinese hamster ovary cells. Mol Endocrinol.

[CR87] Rivas A, Fisher JS, McKinnell C, Atanassova N, Sharpe RM (2002). Induction of reproductive tract developmental abnormalities in the male rat by lowering androgen production or action in combination with a low dose of diethylstilbestrol: evidence for importance of the androgen-estrogen balance. Endocrinology.

[CR88] Roa J, Vigo E, Castellano JM, Gaytan F, Navarro VM, Aguilar E, Dijcks FA, Ederveen AG, Pinilla L, van Noort PI, Tena-Sempere M (2008). Opposite roles of estrogen receptor (ER) alpha and ERbeta in the modulation of luteinizinghormone responses to kisspeptin in the female rat: implications for the generation of the preovulatory surge. Endocrinology.

[CR89] Russell LD, Burguet S (1977). Ultrastructure of Leydig cells as revealed by secondary tissue treatment with a ferrocyanide-osmium mixture. Tissue Cell.

[CR90] Sandner F, Welter H, Schwarzer JU, Köhn FM, Urbanski HF, Mayerhofer A (2014). Expression of the oestrogen receptor GPER by testicular peritubular cells is linked to sexual maturation and male fertility. Andrology.

[CR91] Santolla MF, Lappano R, De Marco P, Pupo M, Vivacqua A, Sisci D, Abonante S, Iacopetta D, Cappello AR, Dolce V, Maggiolini M (2012). G protein-coupled estrogen receptor mediates the up-regulation of fatty acid synthase induced by 17β-estradiol in cancer cells and cancer-associated fibroblasts. J Biol Chem.

[CR92] Sapino A, Pietribiasi F, Bussolati G, Marchisio PC (1986). Estrogen- and tamoxifen-induced rearrangement of cytoskeletal and adhesion structures in breast cancer MCF-7 cells. Cancer Res.

[CR93] Schulster M, Bernie AM, Ramasamy R (2016). The role of estradiol in male reproductive function. Asian J Androl.

[CR94] Sewer MB, Li D (2008). Regulation of steroid hormone biosynthesis by the cytoskeleton. Lipids.

[CR95] Sharma G, Prossnitz ER (2011). Mechanisms of estradiol-induced insulin secretion by the G protein-coupled estrogen receptor GPR30/GPER in pancreatic beta-cells. Endocrinology.

[CR96] Shen M, Shi H (2015) Sex hormones and their receptors regulate liver energy homeostasis. Int J Endocrinol 294278 10.1155/2015/294278 PMC4600502 26491440

[CR97] Shen WJ, Azhar S, Kraemer FB (2016). Lipid droplets and steroidogenic cells. Exp Cell Res.

[CR98] Shenoy SK, Lefkowitz RJ (2011). β-Arrestin-mediated receptor trafficking and signal transduction. Trends Pharmacol Sci.

[CR99] Sirianni R, Chimento A, Ruggiero C, De Luca A, Lappano R, Ando S, Maggiolini M, Pezzi V (2008). The novel estrogen receptor, G protein-coupled receptor 30, mediates the proliferative effects induced by 17beta-estradiol on mouse spermatogonial GC-1 cell line. Endocrinology.

[CR100] Smolen AJ, Conn PM (1990). Image analytic techniques for quantification of immunocytochemical staining in the nervous system. Methods in neurosciences.

[CR101] Thomas C, Gustafsson JA (2011). The different roles of ER subtypes in cancer biology and therapy. Nat Rev Cancer.

[CR102] Thomas P, Dressing G, Pang Y, Berg H, Tubbs C, Benninghoff A, Doughty K (2006). Progestin, estrogen and androgen G-protein coupled receptors in fish gonads. Steroids.

[CR103] Treen AK, Luo V, Chalmers JA, Dalvi PS, Tran D, Ye W, Kim GL, Friedman Z, Belsham DD (2016). Divergent regulation of ER and kiss genes by 17β-estradiol in hypothalamic ARC versus AVPV models. Mol Endocrinol.

[CR104] Vaucher L, Funaro MG, Mehta A, Mielnik A, Bolyakov A, Prossnitz ER, Schlegel PN, Paduch DA (2014). Activation of GPER-1 estradiol receptor downregulates production of testosterone in isolated rat Leydig cells and adult human testis. PLoS One.

[CR105] Vrtačnik P, Ostanek B, Mencej-Bedrač S, Marc J (2014). The many faces of estrogen signaling. Biochem Med (Zagreb).

[CR106] Wang YX, Li M, Zhang HQ, Tang MX, Guo CF, Deng A, Chen Y, Xiao LG (2016). Opposite function of ERα and ERβ in controlling 17β-estradiol-mediated osteogenesis in osteoblasts. Arch Med Res.

[CR107] Zarzycka M, Gorowska-Wojtowicz E, Tworzydlo W, Klak A, Kozub K, Hejmej A, Bilinska B, Kotula-Balak M (2016). Are aryl hydrocarbon receptor and G-protein-coupled receptor 30 involved in the regulation of seasonal testis activity in photosensitive rodent-the bank vole (Myodes glareolus)?. Theriogenology.

[CR108] Zhang X, Ke S, Chen K-H, Li J-H, Ma L, Jiang X-W (2015). Diethylstilbestrol affects the expression of GPER in the gubernaculum testis. Int J Clin Exp Pathol.

[CR109] Zimmerli UU, Hedinger CE (1991). Hyperplasia and hypertrophy of Leydig cells associated with testicular germ cell tumours containing syncytiotrophoblastic giant cells. Virchows Arch A Pathol Anat Histopathol.

